# Advances in CNS PET: the state-of-the-art for new imaging targets for pathophysiology and drug development

**DOI:** 10.1007/s00259-019-04488-0

**Published:** 2019-09-21

**Authors:** Stuart P. McCluskey, Christophe Plisson, Eugenii A. Rabiner, Oliver Howes

**Affiliations:** 1 Invicro LLC, A Konica Minolta Company, Burlington Danes Building, Imperial College London, Hammersmith Hospital, Du Cane Road, London, W12 0NN UK; 2 Psychiatric Imaging Group, MRC London Institute of Medical Sciences, Imperial College London, Hammersmith Hospital, London, UK

**Keywords:** PET, CNS, Brain, First-in-human

## Abstract

**Purpose:**

A limit on developing new treatments for a number of central nervous system (CNS) disorders has been the inadequate understanding of the in vivo pathophysiology underlying neurological and psychiatric disorders and the lack of in vivo tools to determine brain penetrance, target engagement, and relevant molecular activity of novel drugs. Molecular neuroimaging provides the tools to address this. This article aims to provide a state-of-the-art review of new PET tracers for CNS targets, focusing on developments in the last 5 years for targets recently available for in-human imaging.

**Methods:**

We provide an overview of the criteria used to evaluate PET tracers. We then used the National Institute of Mental Health Research Priorities list to identify the key CNS targets. We conducted a PubMed search (search period 1st of January 2013 to 31st of December 2018), which yielded 40 new PET tracers across 16 CNS targets which met our selectivity criteria. For each tracer, we summarised the evidence of its properties and potential for use in studies of CNS pathophysiology and drug evaluation, including its target selectivity and affinity, inter and intra-subject variability, and pharmacokinetic parameters. We also consider its potential limitations and missing characterisation data, but not specific applications in drug development. Where multiple tracers were present for a target, we provide a comparison of their properties.

**Results and conclusions:**

Our review shows that multiple new tracers have been developed for proteinopathy targets, particularly tau, as well as the purinoceptor P2X7, phosphodiesterase enzyme PDE10A, and synaptic vesicle glycoprotein 2A (SV2A), amongst others. Some of the most promising of these include ^18^F-MK-6240 for tau imaging, ^11^C-UCB-J for imaging SV2A, ^11^C-CURB and ^11^C-MK-3168 for characterisation of fatty acid amide hydrolase, ^18^F-FIMX for metabotropic glutamate receptor 1, and ^18^F-MNI-444 for imaging adenosine 2A. Our review also identifies recurrent issues within the field. Many of the tracers discussed lack in vivo blocking data, reducing confidence in selectivity. Additionally, late-stage identification of substantial off-target sites for multiple tracers highlights incomplete pre-clinical characterisation prior to translation, as well as human disease state studies carried out without confirmation of test-retest reproducibility.

## Introduction

Neurological and neuropsychiatric disorders are a major contributor to global disease burden and economic costs [1, 2]. This highlights the importance of identifying the molecular mechanisms underlying them and evaluating novel therapeutic strategies to combat them.

It is well known that drug development programmes are expensive and risky due to low success rates. For therapeutics targeting the central nervous system (CNS), these issues are amplified, with substantially longer average development times and reduced success rate over non-CNS targets, such as those for cardiac or gastrointestinal disorders [3]. One contributor to CNS drug failure is the additional pharmacokinetic challenge of crossing the blood-brain barrier (BBB) [3]. Peripheral measurement of drug concentration is often a poor representation of availability within the CNS; therefore, knowing if a drug has reached the brain in high enough concentrations for pharmacological effect is important [4].

Molecular imaging techniques such as positron emission tomography (PET) have the ability to quantitatively characterise molecular targets and target occupancy, within the CNS. PET utilises short-lived isotopes which decay to emit two gamma photons in approximately opposite directions. The molecular sensitivity of PET imaging, and the capacity to selectively image target-ligand interactions in vivo at tracer doses, gives this technique the ability to probe CNS targets with high selectivity and sensitivity in humans [5]. This information can be utilised to further the understanding of pathologies and identify new targets for therapeutic intervention, allowing innovative strategies to be designed. Additionally, molecular imaging has the ability to characterise the pharmacokinetics and selectivity of CNS-targeted drugs and is now a common strategy in drug development programmes. This allows greater characterisation of investigational drugs, potentially reducing the risk of costly late-stage failure and increasing overall efficiency of drug development programmes.

PET imaging has an advanced understanding of a number of neurological and psychiatric conditions. Some well-known examples include ^18^F-FDG for imaging alterations in glucose metabolism across disease states [6, 7], ^18^F-FDOPA to index dopamine synthesis capacity in Parkinson’s disease (PD) and schizophrenia [8], ^11^C-PIB for tracking the accumulation of amyloid β plaques in Alzheimer’s disease (AD) [9], and multiple tracers for imaging translocator protein in multiple disease states including AD, PD, and Creutzfeldt-Jakob disease [10].

The aim of this review is to provide an overview of recent developments in PET imaging probes for CNS targets in humans and to evaluate the potential of PET imaging tools available. A critical assessment of both the pre-clinical and in-human characterisation of the novel PET tracers is conducted. For each tracer, we summarise the evidence of its properties in terms of criteria for evaluating CNS tracers as tools for the investigation of pathophysiology or target engagement by a drug. Whilst these properties are important for the use of a tracer in drug evaluation, specific applications of tracers for drug development are beyond the scope of this review. Potential confounds of the tracers are discussed, and areas of in vivo characterisation currently lacking in the literature highlighted. Where sufficient data is available, comparisons between tracers are conducted and future potential of both tracer and target proposed.

The cut-off for ‘recent’ was defined as a first peer-reviewed publication from the last 6 years (1st of January 2013 to 31st of December 2018 inclusive). ‘New’ targets were defined as having no in-human PET tracers published for that target prior to this timeframe, or where the tracer(s) published within this timeframe were judged to represent a significant advance over tracers published prior to this period.

## Criteria for evaluating CNS PET tracers and outcome parameters

This section gives an overview of what is required for a CNS PET tracer and highlights the challenges to overcome in the development of a successful tracer. For an in-depth comprehensive review on criteria for CNS PET tracers, refer to the review by Victor Pike (2016) [11].

### Ability to accumulate within the CNS

An obvious requirement for a CNS PET tracer is the accumulation within the CNS; however, achieving this in tracer design is non-trivial. Multiple factors play a key role in determining the success or failure of a tracer in this facet. Some published ‘rule of thumb’ criteria for passive diffusion into the brain are highlighted in Box 1 [11, 12]. High-molecular-weight compounds often struggle to cross the tight junction in the blood-brain barrier leading to no or very slow accumulation within the brain and rendering them unsuitable for PET imaging [11]. The lipophilicity of a compound is essential for accumulation and availability within the brain. This is often determined from the partition coefficient between octanol and aqueous phases at physiological pH, quantified as the LogD_7.4_. If the LogD_7.4_ is too low, then the tracer will be unable to passively cross lipid membranes preventing accumulation in the brain, unless there is active transport. However, if it is too high, the compound will preferentially remain within lipid bilayers, increasing non-specific binding and decreasing the availability and dynamic range.
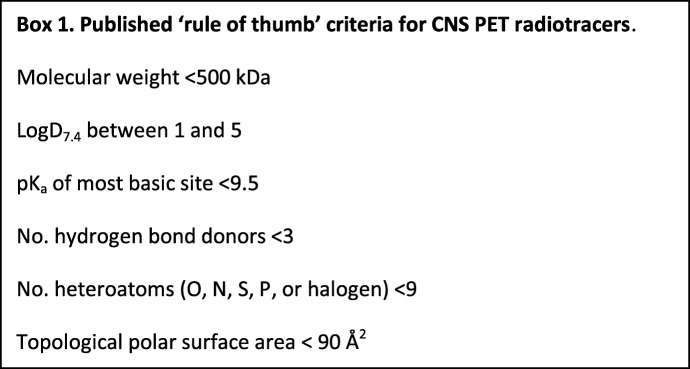


Other parameters such as charge and polarity play a part in the lipophilicity, but have also been linked to increasing susceptibility for being efflux transporter substrates [13, 14]. Efflux transporters are responsible for the inability of a large proportion of drugs and pharmaceuticals to accumulate in the brain, shuttling the compounds back into the bloodstream too fast to allow accumulation [11]. These efflux transporters, which include P-glycoprotein (P-gp), multidrug resistance–associated protein (MDR), and breast cancer–resistant protein (BCRP), vary considerably between species and often render substrates useless for CNS applications [15].

### Suitable pharmacokinetics and selectivity

The ability to cross the BBB is an essential criterion for all CNS PET tracers, but the pharmacokinetics and selectivity ultimately determine a PET tracer’s usefulness. Factors impeding or reducing its ability to accurately report on its target can severely limit its applicability or render it unusable. Some of these key parameters are listed in Box 2.
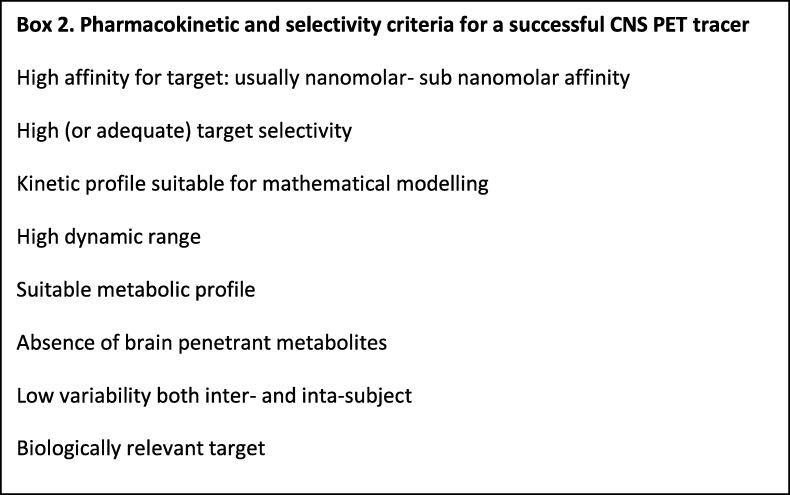


While an ideal PET tracer would be outstanding in all of the criteria listed in Box 2, in practice, this is often unachievable. Fully characterising the limitations of a tracer allows informed decisions on its applicability to answer the proposed question, and importantly when it is not. For example, consider a hypothetical tracer which has overall good properties, but shows additional high off-target binding within the brain. In brain areas where both target and off-target sites are present, this will often render the tracer unable to answer the desired question. However, in brain regions with low or no off-target site, it may be possible to accurately quantify the target and gain an accurate answer. Therefore, if the off-target site is known and well-characterised, then a tracer with off-target binding in some brain regions may, nevertheless, be useful for studies where the focus is the regions with low off-target binding.

### Common outcome parameters

To effectively interpret the results presented in this review, an understanding of the various outcome parameters used in these studies is necessary. Below is an overview of some of the most common outcome parameters used in PET studies, both pre-clinically and in-human, summarised in (Table 1). For a comprehensive review, see reference [11] or refer to the Turku PET centre website (http://www.turkupetcentre.net).

**Table 1:** Basic description of common outcome parameters used in PET imaging studies

Outcome parameter	Full name	Equation	Description
%ID	Percentage injected dose	$\frac{\%\mathrm{of}\ \mathrm{injected}\ \mathrm{dose}}{\mathrm{g}\ \mathrm{of}\ \mathrm{tissue}}$	Percentage of injected radiotracer per unit volume (or weight) of tissue
SUV	Standardised uptake value	$\frac{\%\mathrm{ID}\times 100}{\mathrm{subject}\ \mathrm{weight}}$	Weight corrected parameter of %ID
SUVr	Relative SUV	$\frac{{\mathrm{SUV}}_{\mathrm{target}}}{{\mathrm{SUV}}_{\mathrm{other}}}$	Ratio between SUV values between target and other regions
*V*_T_	Volume of distribution	$\frac{C_{\mathrm{T}}}{C_{\mathrm{P}}}$	Ratio of tracer concentration between tissue and plasma at equilibrium
DVR	Distribution volume ratio	$\frac{V_{\mathrm{T}\ \left(\mathrm{tissue}\right)}}{V_{\mathrm{T}\ \left(\mathrm{ND}\right)}}$	Ratio of *V*_T_ between tissue and non-displaceable tissue component (often given by reference region when available)
BP_ND_	Binding potential non-displaceable	$\frac{V_{\mathrm{T}\ \left(\mathrm{tissue}\right)}}{V_{\mathrm{T}\ \left(\mathrm{ND}\right)}}-1$	Normalised DVR; when *V*_T (tissue)_ = *V*_T (ND)_, BP_ND_ = 0
RO	Receptor occupancy	$\frac{{\mathrm{BP}}_{\mathrm{ND}\ \left(\mathrm{baseline}\right)}-{\mathrm{BP}}_{\mathrm{ND}\ \left(\mathrm{drug}\right)}}{{\mathrm{BP}}_{\mathrm{ND}\ \left(\mathrm{baseline}\right)}}\times 100\%$	% of receptors occupied, usually by drug administration
TRV	Test-retest value	$\left|\frac{{\mathrm{OP}}_{\mathrm{test}}-{\mathrm{OP}}_{\mathrm{retest}}}{\left({\mathrm{OP}}_{\mathrm{test}}+{\mathrm{OP}}_{\mathrm{retest}}\right)/2}\right|\times 100\%$	Average variation of OP from two scans on the same subject without intervention
COV	Coefficient of variance	$\frac{\mathrm{SD}}{\mathrm{Mean}}\times 100\%$	Average variation of OP within a group
ICC	Intraclass correlation coefficient	$\frac{\mathrm{BSMSS}-\mathrm{WSMSS}}{\mathrm{BSMSS}+\left(K-1\right)\times \mathrm{WSMSS}}$	Comparison of reliability of within-subject variability to between-subject variability

SUV_target_ is the standardised uptake value of the target region, SUV_other_ is the standardised uptake value of other regions, *C*_T_ is the concentration of PET tracer in tissue, *C*_P_ is the concentration of PET tracer in plasma, *V*_T (tissue)_ is the tissue volume of distribution, *V*_T (ND)_ is the non-displaceable volume of distribution, BP_ND (baseline)_ is the non-displaceable binding potential at baseline, BP_ND (drug)_ is the non-displaceable binding potential at after drug administration, OP_test_ is the outcome parameter measurement from an initial test scan, OP_retest_ is the outcome parameter measurement from a repeated scan, SD is standard deviation, BSMSS is between subject mean sum of squares, WSMSS is the within-subject mean sum squares, *K*, in this case, is the number of repeated observations.

The most simplistic outcome measures quote the proportion of radiotracer in the target region at a designated time, such as the percentage injected dose per gram of tissue (%ID) or the injected dose corrected for subject weight, standardised uptake value (SUV). Ratios of uptake between areas (SUVr) provide easy to obtain and useful outcome parameters in early characterisation of a PET tracer and can be justified in human studies when assumptions can be made regarding constant radiotracer delivery and brain non-displaceable binding. However, for many tracers and applications in humans, they do not provide sufficient characterisation of the target to be useful. A useful parameter is the ratio of tracer in a target region in comparison with the tracer in the blood plasma. For reversible tracers, this ratio becomes constant at equilibrium and is quoted as the volume of distribution, *V*_T_ [16]. *V*_T_ is commonly used as an outcome parameter in human studies of reversible tracers. As a parameter, *V*_T_ is a measure of both specific (displaceable) and non-specific (non-displaceable) signal and, as such, can be insensitive to change or differences, especially if background signal is relatively high (where a large change in target availability/density may only cause small changes in observed *V*_T_). Additionally, to calculate *V*_T_, blood sampling, and metabolite correction is required, increasing the time, effort, and invasiveness of PET procedures. Correcting for background non-specific binding (non-displaceable binding seen upon blocking the target, denoted ND) across the brain allows a more sensitive measure of target alterations.

Often for a given tracer, regions of the brain contain no or negligible quantities of the target protein and represent only non-specific binding. This region can thus serve as a reference region to account for non-specific binding in the region of interest. As non-displaceable binding is generally assumed to be constant across the brain, the very useful ratio of *V*_T_/*V*_ND_ (DVR) can be easily obtained when a reference region is present. The SUVr between the region of interest and the reference region at equilibrium gives DVR (and BP_ND_) without the necessity of plasma input methods (as plasma component cancels out) [16]. However, this calculation should always be initially validated against full plasma input methods in humans to determine suitability and bias. DVR and BP_ND_ are parameters which are more sensitive to alteration of target availability/density for reversible tracers than other outcomes, with a decrease of 100% BP_ND_ representing full block of the target. As DVR or BP_ND_ are intrabrain comparison outcomes, they give no information on the overall brain uptake of a tracer.

An important parameter to consider in the design of studies is the variability of the measurement, as this influences the sample size required to sufficiently power a study. Variability can be considered as within-subject (measured by test-retest value (TRV)) and between-subjects, which is often expressed as the coefficient of variation (COV). For tracers where these values are high, delineating small alterations in outcome parameters becomes increasingly more difficult as measurement variability obscures effects. As a rule of thumb, alterations in outcome parameters similar or less than inherent variability will not be accurately quantified on small-scale studies. The intraclass correlation coefficient (ICC) is a measure of reliability comparing intra- and inter-subject variability.

Lastly, it is important to remember that multiple factors may contribute to the alteration of an outcome parameter in vivo. Some of these include differences in target expression, alteration in target affinity (i.e. high or low-affinity states), internalisation of a target, and changes in endogenous occupancy [17].

**Table:** Box 3 Abbreviations

TRV Test retest value AD Alzheimer’s disease ALS Amyotrophic lateral sclerosis A_2A_ Adenosine 2A α7-nAChR α-7 subtype of the nicotinic acetylcholine receptor BBB Blood-brain barrier *B*_max_ Target density BP_ND_ Binding potential (non-displaceable) cAMP Cyclic adenosine monophosphate CBD Corticobasal degeneration cGMP Cyclic guanosine monophosphate CN Cognitively normal CNS Central nervous system COV Coefficient of variance COX Cyclooxygenase DLB Dementia with Lewy bodies DVR Distribution volume ratio FAAH Fatty acid amide hydrolase FDA Food and Drugs Administration GABA γ-aminobutyric acid GBq Gigabecquerel HChealthy controls HD Huntington’s disease ICC Intraclass correlation coefficient I2BS Imidazoline 2 subtype binding site KO Knockout LBD Lewy body disorders M Molar MAO Monoamine oxygenase MCI Mild cognitive impairment mGluR Metabotropic glutamate receptor MRI Magnetic resonance imaging MS Multiple sclerosis NFT Neurofibrillary tangles NHP Non-human primate OLR Opioid-like receptor OR Opioid receptor PD Parkinson’s disease PDE Cyclic nucleotide phosphodiesterase PET Positron emission tomography PSP Progressive supranuclear palsy RO Receptor occupancy SPECT Single-photon emission computed tomography SRTM Simplified reference tissue model SUV Standardised uptake value SUVr Relative standardised uptake value SV2A Synaptic vesicle glycoprotein 2A TCM Tissue compartment model TDP-43 TAR DNA-binding protein TRPV1 Vanilloid receptor TSPO Translocator protein VAChT Vesicular acetylcholine transporter V_T_ Volume of distribution Κ-OR Kappa opioid receptor 5-HT Serotonin %ID Percentage injected dose	

## Methods

### Search strategy

The list of tracer and target systems was based on the national institute of mental health research priorities list (https://www.nimh.nih.gov/research-priorities/therapeutics/cns-radiotracer-table.shtml) and supplemented by hand-searching of references, including of recent review articles [18, 19]. PET tracers with the first-in-human peer-review publication between 2013 and 2018 were considered. This was refined with further inclusion criterion of PET tracers for targets that had not previously been imaged in man, or where the tracer(s) published within this timeframe represented a ‘significant advancement’ over those published prior to 2013. A significant advancement was defined as the potential of a tracer to answer questions about the targets that were previously unobtainable or with much greater accuracy. Additionally, PET tracers with first-in-human studies reported at conference between 2013 and 2018, but without associated first-in-human peer-review publication, were also included if other PET tracers for that target met the inclusion criterion of the article.

PubMed literature search terms including (CNS and PET), (brain and PET and first in human), (‘target’ and PET), and (‘target’ and imaging) were used to identify tracers translated into human. Tracers meeting selectivity criteria were systematically reviewed via PubMed literature search of all articles containing the [‘tracer’] term, including previous names and isotopologues, including non-radioactive molecule. The published literature for each in-human tracer was collated and compared with other tracers using the evaluation criteria outlined below.

### Evaluation criteria

In-human tracers were assessed via two categories of criteria: the selectivity of the tracer in vivo and its pharmacokinetic profile. Selectivity was primarily assessed from in vivo blocking and occupancy data. In vitro techniques were also considered, especially in cases where specific off-target sites were investigated. Where a known off-target specific binding site was found for a tracer, discussion of the potential impact on quantification of the desired target is also conducted.

In vitro studies were deemed insufficient to extrapolate to proof of in vivo selectivity. Self-blocking and structurally dissimilar heterologous blocking with selective agents in vivo allow assessment of total specific binding and total selective binding, respectively, at full occupancy. Tracers were deemed to have proven high specificity or high selectivity if the outcome parameter approached saturation value upon relevant blocking experiment. At full occupancy, theoretical alteration of BP_ND_=0, DVR=1, SUVr=1, RO=100%. For situations where full occupancy is unobtainable, i.e. due to toxicity, Lassen plots can provide a suitable alternative; however, they become less accurate at lower occupancies [20]. For parameters without correction for non-displaceable binding, such as *V*_T_ and SUV, the magnitude of decrease depends on the proportion of non-displaceable binding present and therefore is expected to show lower relative alterations than other outcome parameters.

Studies which specifically investigate off-target binding were also investigated and potential impact on tracer assessed. Compounds which bind to sites other than the target site can be used in blocking studies to determine if the tracer also binds to these off-target sites. Additionally, self-blocking experiments where a reduction in signal is observed in regions where no specific signal is expected (i.e. in brain regions where no target is present, in target knockout models, or in healthy controls (HC) tissues not expressing the target of interest) highlight areas of off-target specific binding which can perturb target quantification.

The pharmacokinetic profile of a tracer encompasses multiple parameters essential for tracer performance in vivo. The ability to efficiently cross the BBB is fundamental to a CNS PET tracers’ success. Outcome measures related specifically to brain signal, such as SUV or *V*_T_, were assessed for evidence of this. Further observation of a PET tracers’ regional brain distribution provides circumstantial evidence of selectivity when correlating with known distribution of the target.

The accuracy and reliability of modelling techniques to produce outcome parameters depend partially on the kinetics of a tracer. Slow kinetics generally requires longer scan times and produces more variable outcome measures, reducing the usefulness of a tracer.

The dynamic range of a tracer is the proportion of signal alteration that can occur under a perturbed system (i.e. during an occupancy study or altered expression in disease state). A high dynamic range allows smaller alterations in a system to be accurately detected, thus increasing sensitivity. Tracers with low dynamic range (for example due to high non-specific binding) may not be able to accurately determine even large alterations in target availability, rendering the tracer incapable of quantifying target accurately.

The presence of a reference region, an area of the brain with no or very low specific signal, allows simplified calculation of outcome parameters such as DVR and BP_ND_ without invasive arterial input functions. This allows a simplified scanning procedure, reducing invasiveness and potentially improving the accuracy of outcome parameters. A reference region was deemed validated if there was evidence of a strong correlation between the outcome parameters calculated from full arterial input function and reference tissue methods. Any evidence of consistent bias of reference region models compared to plasma input methods was reported (Tables 2, 3, and 4).

The metabolism of a tracer can play a key role in the success or failure of a tracer. Rapid metabolism in vivo reduces the availability of tracer to bind to the target, reducing signal magnitude. Additionally, the presence of radiometabolites within the brain can have a huge impact on the quantification of the desired target. Evidence relating to rapid metabolism, radiometabolite formation within the brain, or peripheral formation of brain penetrant metabolites is discussed along with potential impact on tracer performance.

Intra- and inter-subject variability was assessed from published in human data quoting TRV and ICC or COV, respectively. Higher variability reduces the usefulness of a PET tracer, requiring larger sample size studies to delineate alterations between populations. Therefore, the clinical utility of tracers with moderate to high variability is more limited than those with low variability. TRV <10%, COV <10%, and ICC >0.8 were deemed as low variability and a high intraclass correlation coefficient, respectively.

Lastly, the radiochemical parameters of the PET tracer can also have an impact on outcome parameters. For human studies, it is assumed that high radiochemical purity is a minimum requirement and is not discussed within. However, the ratio of radioactive tracer to non-radioactive isotopologue can vary substantially between tracers and individual syntheses. The common measurements of this are either specific activity, with units of GBq/μg or molar activity GBq/μmol [153]. As a directly comparable term between tracers and targets, molar activity is used throughout this article. Low molar activity can result in high injected mass of non-radioactive compound and can cause non-negligible self-blocking, with a magnitude dependant on tracer and target.

### Review format

The CNS targets are discussed in three sections: proteinopathies, which focusses on imaging of misfolded protein aggregates; receptors and transporter proteins; and enzymatic targets. The evidence for tracer selectivity and pharmacokinetic parameters is summarised in tables at the beginning of each section along with a short summary of the current advantages and disadvantages of each tracer. Within this table blocking study, results are quoted from the region of highest alteration, at the largest target occupancy dose. Studies with highly structurally related blocking agents are included within homologous blocking studies due to the high likelihood of displacing the tracer from all specific binding sites. Reference regions listed have been validated in humans unless otherwise stated.

Each target is introduced and its relevance in human disease state is summarised. The in-human tracers for the target are discussed collectively, outlining the evidence for selectivity followed by pharmacokinetic suitability. Where tracer limitations or lacking evidence is apparent, this is also highlighted within these sections. When available, results from in-human disease states are also succinctly summarised. Overall evaluation of the target and the available tracers’ applicability for imaging it is summarised in the final discussion section of each target.

## Targets for proteinopathies

Proteinopathy is the abnormal accumulation of misfolded protein. These insoluble aggregates are commonplace in neurodegenerative diseases such as AD and PD and are thought to be the driving factors in pathology [154]. Three major forms of protein aggregates known to contribute to proteinopathy in the human brain are amyloid β plaques, tau, and α-synuclein [154, 155]. There have been a number of well-established PET tracers used for detection of amyloid in humans for well over a decade [156], and as such, amyloid tracers fall out of the scope of this review. In contrast, to date, α-synuclein has no promising in-human PET tracers. The field of tau imaging, however, has erupted in the last 5 years with the emergence of the first widely successful tau tracers and the highest number of tracers progressing into human studies of any CNS target within that time.

## Tau imaging

The aggregation of tau proteins into neurofibrillary tangles (NFTs) is widely associated with AD as a pathologic hallmark [154]. Its accumulation has been shown to correlate with the disease progression and symptoms of the disease [154], with the ‘Braak staging’ of AD based on the spread of NFTs across the brain [157]. Therefore, there is a large dynamic alteration in NFT accumulation from cognitively normal, through to high risk asymptomatic, mild cognitive impairment, and demented patients. As an example, the first published PET study involving autosomal dominant AD showed cognition correlated strongly with tau imaging, while amyloid β concentration increased significantly over cognitively normal controls approximately 15 years prior to the onset of disease, highlighting the importance of both proteinopathies in the AD pathology [158].

Multiple other neurodegenerative diseases besides AD show characteristic accumulation patterns of tau, often referred to collectively as tauopathies. For neuroimaging of AD and other tauopathies, tracers targeting tau are very attractive for diagnosis and staging. However, until recently, no tau imaging agents were available.

The first tracers investigated, including ^11^C-PBB3 and ^18^F-FDDNP, showed an array of restricting issues including low brain uptake, brain-penetrating metabolites, and amyloid β binding, respectively, hindering wide-scale use in humans [159–162]. Since then, from 2013 to 2018, we identified 7 tracers with first-in-human studies, and a further 5 have clinical trials initiated or initial in-human results presented at conferences (Table 2) Of these, ^18^F-AV-1451 and the ^18^F-THK series are the most widely studied [163].

**Table 2:** Summary of PET radiotracers for tau evaluated in humans since 2013

Tracer	First in human	In vivo homologous block (parameter, species)	In vivo heterologous block	Human TRV	Inter-subject variability outcome: value (regions)	Reference region	Highest uptake AD (parameter, region)	Uptake HC (parameter, region)	Advantages	Limitations
^18^F-AV-1451	2013 [21]	−45% (*V*_T_, healthy NHP) [22]	N/A	<10% [23]	ICC, >0.90 [23]	Cerebellum [24]	2.2 (SUV_R_, inferior temporal/cerebellum) [25]	1.2 (SUV_R_, inferior temporal/cerebellum) [25]	Most published tau tracer. High selectivity over amyloid [26]. Fast kinetics [21]. Low TRV. Significant response in distinguishing between disease states [27, 28]. Inverse correlation with cognitive scores in AD [25, 29–32].	Multiple off-target specific binding sites [33–35]. Low affinity for non-AD-type tau [33, 36–38].
^18^F-THK523	2014 [39]	N/A	N/A	N/A	N/A	Cerebellum (not validated) [39]	1.9 (SUV_R_, subcortical white matter/cerebellar cortex) [40]	1.6 (SUV_R_, subcortical white matter/cerebellar cortex) [40]	Significant difference in AD compared with that in HC [40].	High white matter retention [40]. Limited selectivity data available. Only moderate selectivity over amyloid [41]. Replaced by later derivatives.
^18^F-THK5117	2015 [42]	N/A	N/A	N/A	N/A	Cerebellum (not validated) [43]	1.3 (SUV_R_, neocortex/cerebellar grey matter) [42]	1.1 (SUV_R_, neocortex/cerebellar grey matter) [42]	Improved properties over ^18^F-THK523 [44]. Distinguished AD from HC [42].	Limited selectivity data available. High white matter binding [42]. Replaced by later derivatives.
^18^F-THK5317	2016 [45]	N/A	N/A	<10% [46]	ICC, >0.85 (isocortical and subcortical), 0.52 (posterior cingulate cortex) [46]	Cerebellum [45]	1.4 (SUV_R_, limbic region/cerebellar grey matter) [46]	1.2 (SUV_R_ limbic region/cerebellar grey matter) [46]	Significant alterations between AD and HC and significant correlation to cognitive scores [46]. Low TRV.	Limited selectivity data available.
^18^F-THK5351	2016 [47]	N/A	−37–52% (MAO inhibitor, MCI and AD) [48]	N/A	N/A	Cerebellum (not validated) [49]	3.0 (SUV_R_, hippocampus/cerebellar cortex) [47]	2.1 (SUV_R_, hippocampus/cerebellar cortex) [47]	Lowest white matter retention of THK series [50]. Significant differences from HC found in AD, CBD and PSP [49, 51, 52]. Significant correlation to cognitive scores in AD [53].	High MAO-B binding shown in vitro and in vivo [48, 54]. Highly limited for in vivo tau imaging.
^18^F-MK-6240	2018 [55]	<−10% (*V*_T_, healthy NHP) [22, 56]	N/A	Ongoing	ICC, >0.95 [57]	Cerebellum [57]	3.8 (SUV_R_, precuneus/cerebellar grey matter) [57]	1.1 (SUV_R_, precuneus/cerebellar grey matter) [57]	Low displaceable off-target binding. Low binding in HC [57]. High ICC. Strong correlation to cognitive scores in AD [55]. High ICC.	Not assessed for non-AD tauopathies. High binding in some non-NFT regions [55]. Some de-fluorination observed [55].
^18^F-RO-948	2018 [58]	N/A	N/A	<10% [58]	ICC, >0.90 [59]	Cerebellum [59]	2.8 (SUV_R_, inferior parietal lobe/cerebellar cortex) [59]	1.4 (SUV_R_, inferior parietal lobe/cerebellar cortex) [59]	Lead compound of RO- series [58]. Significant differences between HC and AD patients [58]. Low TRV and high ICC.	Fast metabolism in human [58]. Limited selectivity data available. Structural derivative of ^18^F-AV-1451 so may suffer similar drawbacks.
^18^F-MNI-815	2015 (CT) [60]	N/A	N/A	N/A	N/A	N/A	N/A	N/A	N/A	Replaced by ^18^F-PI2620 in Primal Imaging’s clinical trials.
^18^F-GTP-1	2016 (CA) [61]	N/A	N/A	Ongoing	N/A	Cerebellum (not validated) [61]	N/A	N/A	Reported correlation to cognitive scores [61].	No peer-reviewed in vivo data available.
^18^F-AM-PBB3	2017 (CA) [62]	N/A	N/A	N/A	N/A	N/A	N/A	N/A	Lower binding in basal ganglia and thalamus than ^11^C-PBB-3 parent tracer [62].	Off-target binding in choroid plexus [62]. No peer-reviewed in vivo data available.
^18^F-PM-PBB3	2017 (CA) [62]	N/A	N/A	Ongoing	N/A	N/A	N/A	N/A	Lower binding in basal ganglia and thalamus than ^11^C-PBB-3 parent tracer [62].	Off-target binding in choroid plexus [62]. No peer-reviewed in vivo data available.
^18^F-PI2620	2018 (CT) [63]	N/A	N/A	N/A	N/A	N/A	N/A	N/A	Low affinity to MAO-A and B via competition assay [64].	No peer-reviewed in vivo data available.

*CT* denotes commencement of a clinical trial where no in human study has yet been published, *CA* denotes conference abstract, *N/A* indicates no published data are available. Values quoted for in vivo blocking studies represent the region of highest alteration observed in the greatest response protocol. TRV and ICC values represent all regions quoted in the corresponding literature unless stated. For a specific method of ICC calculation, please refer to corresponding literature. Highest uptake AD value represents the largest average of the quoted parameter in reported regions in an AD patient group. Uptake HC value represents the average of the quoted parameter in HC of the same region as quoted for highest uptake AD. Reference region quantification has been validated against full plasma input methodologies unless otherwise stated

All tracers reported in peer-reviewed journals have shown significant differences between AD and non-AD controls in brain areas associated with tau aggregation (Table 2). Additionally, significant direct correlations with cognitive scores have been reported for ^18^F-AV-1451 (in multiple studies) [25, 29–32, 158, 164], ^18^F-THK5351 [53], and ^18^F-MK-6240 [55] (Table 2), bringing these tracers to the forefront for disease staging purposes. Both ^18^F-AV-1451 and ^18^F-THK5351 have been studied in disease states and tauopathies other than AD. Significant differences in ^18^F-AV-1451 distribution between patients with PD and progressive supranuclear palsy (PSP) [27] and AD and dementia with Lewy bodies (DLB) [28] have been shown.

^18^F-THK5351 has shown significant response in both PSP and corticobasal syndrome in comparison with that in controls [51, 52]. In PSP, the SUV_R_ of the midbrain, with cerebellum as the reference region, showed the most pronounced difference from controls and was inversely correlated to cognitive scores [51].

### Selectivity studies for tau agents

#### In vivo

Very limited in vivo blocking data are available for tau tracers (Table 2). Homologous block data has been presented for only two tracers, ^18^F-AV-1451 and ^18^F-MK-6240, and heterologous block only reported for ^18^F-THK5317, with a non-tau agent (probing off-target binding). For ^18^F-AV-1451, self-block in healthy non-human primate (NHP), showed a large decrease in signal. Healthy NHPs are devoid of NFT. Therefore, all displaceable signal from a self-block experiment represents specific binding to off-target sites. The large decrease observed for ^18^F-AV-1451 in healthy NHP suggests substantial off-target binding, which may perturb signal quantification in disease groups [22].

For ^18^F-MK-6240, the decrease upon self-block was minimal and showed the proportion of displaceable off-target binding is small in healthy NHP [22]. ^18^F-THK5351 has been pursued as the leading tracer of the THK series due to favourable pharmacokinetics [50]; however, recent in vivo evidence of off-target binding has emerged. In vivo imaging in mild cognitive impairment (MCI) and AD patients after 10 mg of selegiline, used clinically as an irreversible monoamine oxidase (MAO) inhibitor, reduced brain uptake of ^18^F-THK1351 by 37–52% compared with baseline. The greatest decrease was observed in regions expected to have high MAO-B concentrations, and signal loss was maintained during the third scans 9–28 days later [48]. A substantial portion of signal in AD and MCI patients therefore appears to be specific off-target binding to MAO.

#### In vitro

In vitro and ex vivo binding to tau has been reported in peer-reviewed journals for all published tracers [40, 163, 165]. However, further in vitro blocking studies have also indicated potential off-target binding sites for many of the in-human tau tracers.

Multiple studies have found potential off-target binding sites for ^18^F-AV-1451 including indications that off-target binding may be linked to iron accumulation [34], evidence for off-target binding of ^18^F-AV-1451 to neuromelanin- and melanin-containing cells in the substantia nigra[33] and compelling evidence of high affinity and moderate affinity binding to MAO-A and MAO-B, respectively, using ^3^H-AV-1451 [35]. The latter case is interesting as cold AV-1451 showed no inhibition of MAO-A or MAO-B at a 1-μM concentration during initial screening [166]. This example highlights an important subtlety in PET tracer development, where lack of inhibition or activation (i.e. high IC_50_ value) does not necessarily equate to a lack of binding (i.e. high *K*_D_).

For the leading compound of the THK series, ^18^F-THK5351, a study was carried out on the brain tissue of patients who had received a ^18^F-THK5351 scan while alive. The post-mortem autoradiography study on these brains using ^3^H-THK5351 showed concordant uptake between PET scan and autoradiography data but also showed that the vast majority of ^3^H-THK5351 signal was blocked with MAO-B inhibitor lazabemide [54]. This study is consistent with the in vivo data discussed above and provides strong evidence of off-target binding to MAO-B. MAO-B is prevalent across the entire brain, increases with age, has been proposed as a biomarker for astrocytosis (frequently observed at sites of degenerative lesions), and can have variable availability, such as decreased availability due to tobacco inhalation [167, 168]. Therefore, the prevalence of MAO-B in brain regions central to NFT formation coupled with the high degree of ^18^F-THK5351 binding to it will limit the use and interpretation of this tracer for tau imaging in vivo. Results from other derivatives have yet to be published and may suffer similar MAO binding.

A very recent candidate for tau imaging in humans is ^18^F-RO-948 (also referred to as ^18^F-RO6958948). It was deemed to be the lead candidate of three potential tracers during initial translation into humans (^18^F-RO6958948, ^11^C-RO6931643, and ^11^C-RO6924963) [58]. In vitro data showed a good indication of distribution in post-mortem AD brain regions expected to contain tau, with in vitro AV-1451 blocking studies showing large displacement. However, RO-948 is structurally similar to AV-1451, which has evidence of multiple off-target binding sites. Therefore, ^18^F-RO-948 may display similar off-target binding.

### Pharmacokinetic profiles of tau tracers

^18^F-AV-1451, ^18^F-THK5351, ^18^F-MK-6240, and ^18^F-RO-948 all showed rapid brain delivery and fast kinetics suitable for imaging, with ^18^F-THK5351 having the fastest washout from the cerebellum of the THK series [21, 47, 55, 58]. Where results have been published, inter- and intra-subject variability reported is low (Table 2), meaning the repeatability of the outcome parameter(s) of these tracers within patients is high, and the differences in uptake across HC subjects is low. Additionally, the cerebellum appears a suitable reference region for tau imaging and is validated as such for multiple tau tracers (Table 2).

Retention in areas of the brain not expected to contain tau or in HC can indicate off-target binding and perturb quantification of tau in vivo. ^18^F-AV-1451 shows sites of high uptake in HC with the most prominent being the basal ganglia, mid-brain, and choroid plexus [163]. Initial compounds of the THK series ^18^F-THK523 and ^18^F-THK5117 showed high white matter retention in HC [40, 42], but this was progressively improved over the series, through ^18^F-THK5317 and finally ^18^F-THK5351 [45, 47]. For ^18^F-MK-6240, the ethmoid sinus, clivus, meninges, and substantia nigra had increased uptake, outlining these as sites of off-target binding, as well as some skull uptake, indicative of de-fluorination [55]. Sites with reported off-target binding of ^18^F-RO-948 include the substantia nigra, cerebellar vermis, meninges, and in the retina [58]. The presence of tracer retention in brain areas of HC raises issues of tracer quantification. A high off-target signal will reduce the relative dynamic range of the tracer in that area and may differ in magnitude between individuals or patient groups, making correction for off-target binding difficult. In extreme cases, signal from areas adjacent to sites of high off-target binding may be perturbed due to partial volume effect. Therefore, for example, white matter retention is a substantial barrier to quantification due to widespread distribution and variability between patients. However, for areas removed from expected distribution and spread of tau, such as the retina, or regions of uptake which remain constant across study groups may not present substantial quantification issues.

Several additional tau tracers have been translated into humans and presented at conference including ^18^F-GTP-1 [61], ^18^F-PI-2620 [169] (replacing weaker candidate ^18^F-MNI-815, which has also been translated into humans [60]), ^18^F-AM-PBB3, and ^18^F-PM-PBB3 (also known as ^18^F-MNI-958) (Table 2) [62]. The initial presented data from these tracers appears promising. However, no in vivo data have been published in peer-reviewed journals, preventing objective comparison with the more established tracers. Therefore, the field eagerly awaits the emergence of clinical trial data and associated publications.

### Conclusions and outstanding issues for tau imaging

The large number of recent tracers for tau highlights the impetus associated with the development and translation of tau PET probes within the medical imaging community. The ability to distinguish AD patients from HC and people with MCI on the basis of tau load has been shown in multiple studies. Expansion of tau imaging into other disease states and clinical populations is well underway. Over 60 ‘PET + tau’ clinical studies are currently active or recruiting to investigate tau load in many disease states including dementias, motor neuron diseases, traumatic brain injury, and depression, amongst others (clinicaltrails.gov, data obtained 30 October 2018). There are currently no promising in-human tau-based therapeutics. The ability to track tau load within human subjects is of huge importance for tau-based drug development programmes. Assessment of an anti-tau drugs effect on tau load in vivo, and direct comparison to cognitive performance/disease progression, would provide invaluable information of a drug’s efficacy and the merits of tau reduction as a therapy in humans.

Of all in-human tau tracers, ^18^F-MK-6240 is currently the most promising. The selectivity profile, to date, is the most robust, with minimal off-target specific binding apparent in NHP, and strong correlation to cognitive scores in AD reported. However, for this, and all tau tracers published, there are still many open questions to be addressed. In the following section, we discuss the issues that would be useful to address.

#### Tau has multiple targets

Pathologic accumulation of tau into paired helical filaments and subsequently NFTs initially provide distinct targets. However, intracellular NFTs can be varied in composition and form. Different isoforms of NFT tau are associated with different disease states, predominantly 3R tau in Pick’s disease, 4R in PSP, corticobasal degeneration (CBD) and argyrophilic grain disease, and a 1:1 mix of 3R and 4R in AD [170]. Additionally, the morphology of the NFT is altered between disease states, with AD being characterised by flame-shaped NFT and neuropil threads [170]. As such, different binding sites, and the affinities of tau tracers for them, may vary substantially between isoforms and morphologies of tau. For example, multiple studies have reported that the binding affinity for ^18^F-AV-1451 may be substantially lower for non-AD type tau, restricting its use in imaging other tauopathies [33, 36–38]. Therefore, categorisation of tracers as ‘tau imaging agents’ may be misleading. Screening across diverse tauopathies in vitro would allow assessment of tracers’ affinity across different tau isoforms and the disease states they are most suited to image. For example, ^18^F-AV-1451 may be more aptly described as a ‘3R + 4R tau’ imaging agent.

#### In-depth characterisation of tracers pre-clinically

The current leading tau tracers in terms of published research and inclusion in clinical trials are ^18^F-AV-1451 and ^18^F-THK5351. The rapid translation into clinical trials of these tracers appears to have left a vacuum of in vitro and in vivo data, which is only now catching up. As such, the selectivity profile for these tracers, and all tau tracers discussed, is far from complete. Critical selectivity data for ^18^F-AV-1451 and ^18^F-THK5351 has only emerged after the commencement of multiple large-scale trials. For ^18^F-THK5351, MAO-B binding appears a major confound given the extent of in vivo binding and distribution of MAO and may severely restrict the use of ^18^F-THK5351 as a tau imaging agent. Off-target binding in ^18^F-AV-1451 appears to present issues also, however, appears less substantial than for ^18^F-THK5351.

Conducting in vivo blocking studies in disease models and HC is standard practice for CNS PET tracer development as a measure of selectivity. For proteinopathies, preclinical models rely on a transgenic mouse to induce protein dysfunction. Unfortunately, many current models fail to replicate the type of tau observed in human diseases, with tracers showing much lower binding to murine models [40, 171]. The higher cost of transgenic strains and the caveats of these models may be contributors to the lack of published data available in this area.

Blocking studies in HC, as well as in more representative tau-accumulating disease models, would be a substantial addition to the knowledge of tracer selectivity and dynamic range.

#### Lack of selective compounds

Currently, there are no well-characterised, highly selective compounds available for use in competitive binding studies, preventing the determination of a tracer’s tau specific signal and dynamic range. Development of such agents would allow more robust characterisation of selective tau agents and allow determination of binding site(s) occupied by tracers.

Addressing these challenges is vital for the research effort into imaging of tau and to support research of AD and other tauopathies.

## Receptor, transporter, and synaptic targets

Many neuropsychiatric conditions are thought to be due to or characterised by dysfunction in neuroreceptors, transporters, or synaptic proteins [172]. Additionally, multiple targets within this section are linked to degenerative neurological disorders. PET tracers for these targets are therefore of great importance for characterisation of a wide range of diseases, diagnosis, and drug development programmes [173]. Table 3 highlights the recent tracers for receptor, transporter, and synaptic targets that have been translated into humans from 2013 to 2018.

**Table 3:** Parameters of PET radiotracers for new receptor, transporter, and synaptic targets in humans

System	Target	Tracer	First in human	In vivo homologous block (parameter, species)	In vivo heterologous block (parameter, species)	Human TRV	Interpatient variability outcome: value (regions)	Highest uptake (parameter, region)	Reference region	Advantages	Limitations
Cholinergic	VAChT	^18^F-FEOBV	2014 [65]	−28% (SUVr, rat) [66]	N/A	Ongoing	COV, 20% (striatum) 6–12% (cortical) [65]	25 (BP_ND_, striatum) [65]	Cerebellar grey matter [65]	Improved signal-to-noise over previous SPECT VaChT agents [65]. Significant correlation to cognitive symptoms in AD [67].	Slow kinetics [65]. Only partial homologous block achieved [66]. No heterologous block reported.
		^18^F-VAT	2018 (CA) [68]	N/A	−90% (SUVr-1, NHP [non-selective block]) [69]; −54% (*V*_T_, NHP [non-selective block]) [70]	N/A	N/A	N/A	N/A	Good response to block, moderate kinetics in NHP [69, 70].	Heterologous blocking agent also binds to sigma binding sites.[71] No in-human data published.
	α7-nAChR	^18^F-ASEM	2014 [72]	40% (RO, NHP) [73]	≈−90% (%ID, rat), ≈−80% (*V*_T_, NHP) [74]	11.7±9.8% [75]; 10.8±5.1% [72]	COV, 21.1–27.2% [72]	22 mL/cm^3^ (*V*_T_, putamen) [72]	None available	Good response to blocking experiments [73, 74]. Suitable pharmacokinetics [73]. Used successfully in schizophrenia occupancy study [75].	Target has no reference region.
Adenosine	A2A	^18^F-MNI-444	2015 [76]	100–103% (striatal RO, NHP) [77]	95% (striatal RO, NHP) [77]	<10% on average [76]	COV, 12.2–25.0% (basal ganglia structures) [76]	3.3 mL/cm^3^ (*V*_T_, Putamen) [76]	Cerebellum [76]	High selectivity [77]. Good pharmacokinetics, low background, slow metabolism, low TRV [76]	Small bias with using cerebellum as reference region [76]. Moderately long scan times may be necessary.
Synaptic vesicle proteins	SV2A	^18^F-UCB-H	2015 [78]	N/A	−44% (*V*_T_, whole brain, rat), [79]	N/A	COV, 12.2% (whole brain) [78]	7.8 mL/cm^3^ (*V*_T_, gyrus rectus) [80]	None available	^18^F allows greater availability than ^11^C.	Lower sensitivity and BP_ND_ compared with ^11^C-UCB-J [81]. Target has no reference region.
		^11^C-UCB-J	2016 [82]	−75% (*V*_T_, NHP) [83]	−78% (*V*_T_, NHP) [83]	<10% [84]	ICC, typically >0.6 [84]	23 mL/cm^3^ (*V*_T_, centrum semiovale) [81]	None available	Field leading SV2A tracer [81]. Good selectivity [83]. Fast kinetics, low TRV [84]. Significant alterations in epileptic patients [82].	Target has no reference region.
Imidazoline receptors	I2BS	^11^C-BU99008	2018 [85]	N/A	−53% (ex vivo SUV, rat) [86]; ≈−90% (SUV, NHP) −80% (*V*_T_, NHP) [87]; ≈−60% (whole brain *V*_T_, human) [85]	5–25% [85]	COV, 17.6–31.1% (subcortical structures) [85]	106 mL/cm^3^ (*V*_T_, striatum) [85]	None available	Only tracer for I2BS in humans. Good response in blocking studies [85–87].	Areas of high TRV and COV, slow kinetics, long scan times required [85]. Target has no reference region.
Metabotropic glutamate receptors	mGluR1	^11^C-ITMM	2013 [88]	−85% (SUV, rat) [89]	−85% (SUV, rat) [89]	N/A	COV, <10% (all reported regions except for flocculus), 26% (flocculus) [90]; ≤10% (anterior lobe, posterior lobe vermis) [91]; <20% (multiple cortices) [92]	2.6 mL/cm^3^ (*V*_T_, cerebellar cortex) [88]	White matter (not validated) [93]	Very good in vivo block response [89]. Significant differences found in cerebellar ataxia patients [90, 93]	Relatively low brain uptake, slow kinetics [88]. No validated reference region.
		^18^F-FIMX	2016 [94]	−85% (SUV, NHP) [95]	≈−100% (displacement SUVr, NHP) [95]	Ongoing	COV, 9.4–13.2% [94]	11 mL/cm^3^ (*V*_T_, cerebellum) [94]	N/A	Very good in vivo block response [95]. Fast kinetics, high brain uptake [94].	Fast metabolism in human [95]. No validated reference region.
Opioid	κ	^11^C-GR103545	2014 [96]	Not suitable due to toxicity. [97]	−75% (*V*_T_, human [non-selective block]) [96]	8–41% [96]	ICC, 0.81±0.08 [96]	28 mL/cm^3^ (*V*_T_, amygdala) [96]	None available	Highest affinity for *κ* in vitro [96].	No suitable reference region. Slow kinetics, high TRV in amygdala, low injectable mass tolerance [96].
		^11^C-LY2795050	2014 [98]	−60% (DVR, NHP) [99]	−100% (BP_ND_, NHP) [99]; −59% (*V*_T_, human) [100]	≤10% [101]	ICC, >0.8 (all reported regions except amygdala), 0.56 (amygdala) [101]	4.0 mL/cm^3^ (*V*_T_, amygdala) [100]	None available	Suitable pharmacokinetics for imaging [98]. Good response to *in vivo* blocking [99]. Low TRV.	No suitable reference region. Moderate selectivity over μ opioid receptor in vivo [102]. Limited dynamic range [99].
Serotonin	5-HT2	^11^C-Cimbi-36	2014 [103]	N/A	−64% (BP_ND_, human, non-selective block) [103]; −56% (*V*_T_, NHP, non-selective block) [104]	<10% on average [105]	ICC, 0.72–0.91 (all reported regions except subsequent), 0.24-0.32 (anterior and posterior cingulate and striatum) [105]	40 mL/cm^3^ (*V*_T_, medial inferior temporal gyrus) [103]	Cerebellum (negative bias) [103]	Good response to in vivo block [103, 104]. High uptake in cortical regions, possibility to report on 5-HT2A and 5-HT2C simultaneously [103]. Low TRV	Not selective across 5-HT subfamily [104]. Moderately slow kinetics, bias from using reference region [103]. Low ICC in some regions.
Purinoceptor	P2X7	^18^F-JNJ-64413739	2018 [106]	93% occupancy (HC) [106]	N/A	10.7±2.2% [106]	ICC, >0.90 (2TCM) [106]	3.3 mL/cm^3^ (*V*_T_, brainstem) [106]	None available	First P2X7 tracer published in man. Successful occupancy study conducted [106].	Relatively high COV (33.5±2.2%) [106]. No characterisation with structurally dissimilar block. No suitable reference region.
		^11^C-SMW139	2018 (CT)	N/A	≈−100% (SUVr-1, rat viral vector model) [107]	N/A	N/A	N/A	N/A	Good response to block in rodent model [107].	Limited data available. High non-parent metabolite in rodent brain [107]
		^11^C-JNJ-54173717	2018 (CA) [108]	≈−100% (SUVr-1, rat viral vector model) [109]	≈−65% (SUV, NHP) [109]	N/A	N/A	N/A	N/A	Good response to block in rodent model and in NHP [109].	Limited data available, higher baseline signal in non-hP2X7 areas, possibility of off-target binding [109].
		^11^C-GSK1482160	2018 (CA) [110]	≈−60% (*V*_T_, mouse lipopolysaccharide model) [111]	N/A	N/A	N/A	N/A	N/A	Good response to lipopolysaccharide model induction and block [111]. Promising in vitro and ex vivo response [111, 112]	No characterisation with structurally dissimilar block, slow kinetics, low brain uptake [112]

*CT* denotes commencement of a clinical trial where no in human study has yet been published, *CA* denotes conference abstract, *N/A* indicates no published data is available. Values quoted for in vivo blocking studies represent the region of highest alteration observed in the greatest response protocol. TRV and ICC values represent all regions quoted in the corresponding literature unless stated. For a specific method of ICC calculation, please refer to corresponding literature. Highest uptake value represents the largest average of the quoted parameter in reported regions in HC. Reference region quantification has been validated against full plasma input methodologies unless otherwise stated

## Cholinergic targets

The cholinergic system has been widely related to cognitive decline in disorders including AD, PD with dementia, and Lewy body disorders (LBD), often in tandem with dopaminergic dysfunction [174]. In AD, initial post-mortem data lead to the cholinergic hypothesis which proposes a causal role of reduced acetylcholine synthesis in disease propagation. As a consequence, multiple cholinergic-based treatment strategies to reduce neuropsychiatry symptoms have been developed, with some, but limited, effects [175]. While this hypothesis has fallen in popularity, in place of the amyloid and tau hypotheses, imaging studies have shown a link between the cholinergic system and these pathologies in vivo [174, 176]. Recent MRI studies have provided evidence that basal forebrain pathology precedes and predicts both entorhinal pathology and memory impairment in AD, implicating cholinergic neuronal loss as an early indicator of the disease [177, 178].

As with many neurotransmitters, there are multiple potential targets for cholinergic imaging. Recently, PET tracers for the cholinergic targets of vesicular acetylcholine transporter (VAChT) and alpha-7 subtype of the nicotinic acetylcholine receptor (α7-nAChR) have been reported and characterised.

VAChT activity has been seen as a purer indication of presynaptic cholinergic terminal density than other targets [179] and is distinct from the therapeutic site of cholinesterase inhibitors. [^123^I]IBVM is well-established for imaging and has been utilised in humans for decades [180, 181]. The recently translated ^18^F-FEOBV and ^18^F-VAT potentially offer the inherent benefits of PET tracers over established SPECT tracers, such as higher resolution. For ^18^F-FEOBV, authors claim it allows quantification of VAChT in smaller brain regions, infeasible with ^123^I-IBVM [65].

The α7-nAChR is a cholinergic receptor of great interest across multiple fields. It has been associated with decreased expression in post-mortem schizophrenia tissue [182–184], in traumatic brain injury models [185, 186], and the hippocampus of post-mortem AD tissue [183], as well as increased expression in perirhinal cortex and hippocampus of bipolar post-mortem tissue [187]. Multiple PET tracers have been developed for this target but have failed to perform either pre-clinically or in human studies [188, 189]. The recent translation of ^18^F-ASEM into humans represents the first promising α7-nAChR imaging agent for this target with encouraging performance both pre-clinically and in clinical trials.

### Selectivity data for cholinergic tracers

The lack of in vivo blocking data represents a major drawback for the confidence of selectivity for ^18^F-FEOBV in vivo. In rodents, dose escalation of homologous blocking studies was deemed unethical due to adverse effects of blocking VAChT in vivo; therefore, only a partial self-block was achieved [66]. No heterologous blocking studies have been reported for ^18^F-FEOBV in vivo.

In contrast, large displacement of ^18^F-VAT was observed upon administration of vesamicol in NHP (Table 3) [69, 70]. Vesamicol is not selective for VAChT with well-known binding to sigma receptors [71] and therefore represents a non-selective block. The in vitro characterisation of ^18^F-VAT determined high selectivity of VAT over sigma receptors [69], but this cannot be assumed to translate into in vivo selectivity of a radiotracer, as discussed with ^18^F-AV-1451 above. Nevertheless, substantial binding to sigma seems unlikely as no decrease in SUV was observed in the cerebellum upon blockade, where sigma receptors are prevalent, as well as showing contradictory distribution [190].

For imaging α7-nAChR with ^18^F-ASEM, substantial reduction in uptake upon heterologous α7-nAChR specific block, with DXMB-A and SSR180711 in rodent and NHP respectively has been shown [74, 191], and no response to multiple negative control blocking studies in rodents [74]. These studies imply high selectivity and dynamic range of ^18^F-ASEM for α7-nAChR in vivo.

### Pharmacokinetic properties of cholinergic tracers

^18^F-FEOBV, ^18^F-VAT, and ^18^F-ASEM all show good peak brain activity, heterogeneous distribution in line with target distribution, and moderate to fast kinetics in most brain regions [65, 73]. The exception is the slow kinetics of ^18^F-FEOBV in basal ganglia structures requiring long scan durations or delayed scanning protocols for quantification in this region [65]. Outcome parameter variability interpatient was moderate for ^18^F-FEOBV and ^18^F-ASEM, as well as moderate intrapatient variability reported for ^18^F-ASEM (Table 3). Peer-reviewed in-human, ^18^F-VAT data is not yet available.

As α7-nACh is present across the entire brain, there is no available reference region for ^18^F-ASEM, and therefore, full plasma input methods will be required for accurate quantification. For ^18^F-FEOBV, the cerebellar grey matter has been validated as a suitable reference region and provided lower variability than arterial input measures, with high correlation, however, had a lower dynamic range [65]. In NHP, the cerebellum may also provide a reference region for ^18^F-VAT quantification [70].

### Conclusions and outstanding issues for recent cholinergic tracers

^18^F-FEOBV and ^18^F-ASEM both show pharmacokinetic profiles suitable for imaging and have been successfully utilised in disease state imaging. ^18^F-FEOBV was shown to have the highest sensitivity compared with both ^18^F-FDG (metabolism) and ^18^F-NAV4694 (amyloid) for distinguishing between HC and AD patients in a small-scale study [67]. ^18^F-ASEM *V*_T_ was found to be significantly decreased in schizophrenia patients in some brain regions, although an outlier was excluded in order to achieve this [75]. Additionally, an occupancy of up to 49% was determined when schizophrenic patients were treated with α7-nAChR selective agonist DXMB-A. The significant alteration observed with these tracers in disease state clinical trials shows the prospective applicability of these cholinergic targets in disease state imaging.

The lack of reference region available for α7-nACh increases the practical complexity of scanning with ^18^F-ASEM as quantification will require full arterial input function. The slow kinetics of ^18^F-FEOBV in the basal ganglia structures may result in higher demands on equipment for quantification in these regions, due to long scan duration. The in vivo selectivity data for ^18^F-ASEM appears comprehensive and robust, whereas further work is required for ^18^F-FEOBV.

## Adenosine A_2A_

A large number of human CNS disease studies have implicated adenosine 2A receptors (A_2A_), including schizophrenia [192], Pick’s disease [193], MCI [194], bipolar disorder [195], and HD [196], and additionally in pre-clinical models of AD [197], addiction [198], aging [199], attention deficit hyperactivity disorder [200], epilepsy [201], hyperoxia [202], multiple sclerosis (MS) [203], PD [204], restless leg syndrome [205], and tauopathy [206], amongst others. For in-depth reviews, refer to Waarde et al. [207] and Cheffer et al. [208].

Multiple tracers have been developed for A_2A_ within the CNS, and until recently, the most suitable were ^11^C-TMSX and ^11^C-SCH442416 [209, 210]. Although these tracers are able to cross the BBB and bind to A_2A_, both suffer from high levels of background non-specific binding in human and therefore have a low dynamic range for receptor occupancy studies. As such, studies investigating alterations in A_2A_ availability in the striatum, where A_2A_ is highest, have shown significant alterations [210]. However, studies interested in other regions of the brain are likely to be severely impaired due to the low specific signal to background ratio. The recent development of ^18^F-MNI-444 represents a welcome development with the potential to allow improved characterisation of this interesting target across brain regions.

### Selectivity data of ^18^F-MNI-444

Blocking experiments in NHP with two A_2A_ antagonists, preladenant (structurally similar to ^18^F-MNI-444) and tozadenant (structurally dissimilar to ^18^F-MNI-444), showed a dose-responsive decrease in SUV, with TAC in striatal regions similar to that of the cerebellum at maximum dose for both blocking agents (RO ≈100%) [77]. This represents approximately full blockade of specific signal in these regions providing strong evidence of high A_2A_ selectivity. Preladenant was treated as a homologous block due to the high structural similarity to ^18^F-MNI-444.

### Pharmacokinetic properties of ^18^F-MNI-444

In both NHP and human brains, ^18^F-MNI-444 distribution is heterogeneous and matched that of known A_2A_ receptor distribution [76, 77], with high retention in the striatum and fast washout in the cerebellum, where the concentration of A_2A_ is very low [211]. A limitation is the relatively slow kinetics which may require long scan durations. In blocking studies, a small (<15%), non-dose-responsive decrease in cerebellar activity was observed, making this a non-perfect reference region. However, comparison between arterial input function and reference region analysis methods showed a high correlation, indicating cerebellum may be able to be used as a reference region in both human and NHP, with minimal error [76, 77]. Utilising this method, high intrapatient repeatability was observed [76].

### Conclusions and outstanding issues for ^18^F-MNI-444

There are currently no published results in patients with ^18^F-MNI-444. However, it appears superior for imaging A_2A_ in the CNS to the other evaluated tracers, with a robust selectivity profile and suitable pharmacokinetics for imaging A_2A_. Further studies with ^18^F-MNI-444 are currently underway and will help determine its full potential.

## Synaptic vesicle glycoprotein

The emergence and now widespread use of effective anti-epilepsy medications, such as Brivaracetam [81], which modulate the function of the synaptic vesicle glycoprotein 2A (SV2A) protein, identified this protein as an interesting target. The ubiquitous expression of SV2A in synaptic terminals throughout the brain also brings additional interest as a marker for synaptic density. As such, although this protein is of primary interest to epilepsy, it may also be of use in neurodegenerative diseases where synaptic loss is associated with cognitive impairment [212, 213] and other diseases, such as schizophrenia and depression, where regional synaptic alterations have been implicated [214, 215].

The recent emergence of successful radiotracers for SV2A occurred with the publication of ^11^C-UCB-J and ^18^F-UCB-H in 2014 [216].

### Selectivity data for SV2A tracers

Both ^18^F-UCB-H and ^11^C-UCB-J show a substantial reduction in *V*_T_ upon heterologous block experiments in rat and NHP, respectively, with similar reduction observed with homologous blocking of ^11^C-UCB-J (Table 3). Additionally, screening of ‘cold’ UCB-H and UCB-J showed no activity (<50% effect or inhibition at 10 μM) across a wide range of brain receptors, transporters, enzymes, and ion channels in vitro [79, 83]. This data supports a high degree of SV2A-specific binding and large dynamic range for both ^18^F-UCB-H and ^11^C-UCB-J in vivo.

### Pharmacokinetic profile of SV2A tracers

Due to the near-ubiquitous nature of SV2A across the brain, no suitable reference region is clearly established, although the centrum semiovale is being evaluated as a pseudo-reference region [217]. Both ^11^C-UCB-J and ^18^F-UCB-H have fast kinetics. However, ^11^C-UCB-J has a higher dynamic range and significantly higher BP_ND_ in NHPs and human and has calculated target density (*B*_max_) and *K*_d_ closely matching that of ex vivo data [81, 82]. As such, ^11^C-UCB-J has been pursued as the lead tracer in this series and showed high stability of *V*_T_ in HC test-retest scans, although ICC values were low indicating high interpatient variability (Table 3) [84].

### Conclusions and outstanding issues for SV2A tracers

Of the two tracers, ^11^C-UCB-J appears to be the superior SV2A tracer, with higher affinity in vivo. However, for distribution purposes, the longer half-life of ^18^F will make ^18^F-UCB-H the option available to sites without an on-site cyclotron. One disease state imaging proof of concept study with the SV2A tracers has been carried out. Patients with medically refractory temporal lobe epilepsy showed higher levels of ^11^C-UCB-J asymmetry (>50%) in the hippocampus compared with that usually observed for ^18^F-FDG (<20%), with controls showing little asymmetry [82]. Direct head-to-head studies on larger cohorts are required; however, this is a very promising initial study into the use of ^11^C-UCB-J in epilepsy. Multiple investigations are ongoing into other diseases using SV2A *B*_max_ as an index for synaptic density. While a decrease in ^11^C-UCB-J signal may represent a loss in synaptic density, it is important to remember that alterations in synaptic vesicle concentration or SV2A regulation or availability may also cause alterations in signal without necessarily correlating to synaptic density and will need to be explored.

Nevertheless, SV2A imaging is one of the most exciting new areas for CNS imaging with scope in many disease states. Clinical trials are currently active in AD and addiction (clinicaltials.gov, accessed 07 November 2018) as well as schizophrenia [218].

## Imidazoline 2 binding site

Imidazoline 2 subtype binding site (I2BS) is distributed across the brain and has been studied in relation to multiple disease states. I2BS has been implicated in rodent models of depression [219, 220], as well as post-mortem reports of significant alteration in AD, heroin addicts, and suicide victims, compared with HC [221–223]. However, the use of non-selective drugs and tracers (such as clonidine and idazoxan) were used for these studies. A post-mortem study on PD and HD tissue with a selective I2BS agonist, ^3^H-2-BFI, found significant increase and decrease in receptor density, respectively [224].

Elucidation of the biology behind these alterations of the I2BS remains an unmet challenge. The development and translation into man of the first I2BS-specific PET tracer ^11^C-BU99008 this year may provide a powerful tool in drug development and studies of this target in neuropsychiatric disorders.

### Selectivity data for ^11^C-BU99008

Ex vivo and in vivo studies with, structurally dissimilar, I2BS selective ligand BU224 showed large decrease in SUV of ^3^H-BU99008 in rats and ^11^C-BU99008 in NHP [86, 87]. The spatial colocalisation and reported affinity of imidazoline ligands for MAO identify this as a potential high-risk site for off-target binding [225]. No significant alteration was observed in NHP when treated with MAO-A and MAO-B ligands, suggesting off-target binding to MAO is negligible [87]. The majority of signal in NHP appears to be due to specific binding to I2BS.

### Pharmacokinetic profile of ^11^C-BU99008

Distribution of ^11^C-BU99008 within the brain followed similar patterns throughout species with basal ganglia structures > cortex > cerebellum, in line with I2BS expression [85]. The kinetics of the tracer are relatively slow, requiring long scan durations in humans, with greater implications to image quality for a ^11^C-labelled tracer due to the shorter half-life. Partial blockade with non-selective I2BS in humans reduced SUV across all brain regions showing lack of available reference region [85].

Test-retest variability in humans showed some discord with variation ranging from 5 to 25% across regions with higher variations found in high uptake regions [85]. The authors postulated the slow kinetics of ^11^C-BU99008 and, therefore, high V_T_ could be a contributing factor. Another possible factor could be the relatively low and highly variable molar activity used (quoted as 35.3±17.5 GBq/μmol), with test scans having over twice the injected mass on average than re-test (3.8±3.2 μg and 1.8±1.1 μg, respectively) [85]. A study in rats investigated the effect of ^11^C-BU99008 molar activity, finding significant increase (+28%) in hypothalamus SUV area under curve integrals using ultra-high molar activity ^11^C-BU99008 (>5000 GBq/μmol) in comparison with standard molar activity samples (55–220 GBq/μmol) [226]. Therefore, the high variability test-retest, and higher average uptake in the retest scans, may be due in part to the molar activity of the radiotracer range used. It should be noted that the method used to produce ultra-high molar activity ^11^C-BU99008 had an order of magnitude lower radiochemical yield than conventional methods.

### Conclusions and outstanding issues for I2BS imaging

^11^C-BU99008 appears to be a selective tracer for I2BS with appropriate properties for imaging in vivo. However, further studies on the dynamics of the tracer, the biology of the target, and relevance in disease states are required. Factors which may improve the reproducibility of *V*_T_ measurements include higher (and more consistent) molar activity of ^11^C-BU99008, a tracer with faster kinetics, or a ^18^F-labelled tracer.

## Metabotropic glutamate receptor 1

As the major neurotransmitter at excitatory synapses within the brain, glutamate and glutamate receptors are of high interest across many neuropsychiatric fields. The metabotropic subclass of glutamate receptors is further categorised into 8 known receptor targets (mGluR1-8). Of these, mGluR5 has multiple tracers reported in the literature prior to the scope of this review [227]; mGluR1 has 2 reported successful tracers translated into man (^11^C-ITMM and ^18^F-FIMX, discussed herein), but the other 6 subtypes lack tracers for human use to date.

Post-mortem expression of mGluR1 has been reported to be significantly altered in DLB (+61%) [228] and schizophrenic hippocampus (−33%) [229], as well as multiple mutations to the mGluR1-encoding gene found regularly in schizophrenia [230, 231]. Negative allosteric modulators of mGluR1 have received recent interest as therapeutics showing efficacy in pre-clinical in vivo models including addiction [232], epilepsy [233], neuropathic pain [234], depression [235], and PD [236].

### Selectivity data for mGluR1 tracers

^11^C-ITMM and ^18^F-FIMX are the two PET tracers successfully translated into humans for mGluR1 and are structurally related. Pre-clinical characterisation provides strong evidence of selectivity for both tracers, with large decrease in signal and removal of brain heterogeneity upon both heterologous (JNJ-16259685 [237]) and homologous blocking protocols [89, 95]. Additional characterisation of ^11^C-ITMM in mGluR1 KO mice showed substantial decrease in uptake compared with wild-type, although only whole-brain data of knockouts, rather than individual regions, were reported [89]. No decrease in uptake of ^18^F-FIMX upon administration of mGluR5 selective ligands was observed, showing a lack of specific binding to this site [95].

The robust pre-clinical characterisation of ^11^C-ITMM and ^18^F-FIMX shows a high level of selectivity for mGluR1 in vivo and gives confidence in these tracers for subsequent studies.

### Pharmacokinetic profile of mGluR1 tracers

Both ^11^C-ITMM and ^18^F-FIMX show brain distribution in humans concordant with mGluR1 expression; however, pharmacokinetic profiles of the tracers differ substantially. ^11^C-ITMM has relatively slow kinetics, requiring long scan times, relatively low brain uptake, and slow metabolism [88]. In contrast, ^18^F-FIMX has higher brain uptake, fast kinetics, and much faster decrease in plasma parent fraction [94]. Currently, neither tracer has published TRV, although for ^18^F-FIMX, it has been outlined as an outcome of a current clinical trial (clinicaltrials.gov, trial identifier: NCT02230592). The pons and the medulla are areas of low mGluR1 expression and have been proposed for use in reference region models [93], although no reference region has been validated in humans to date.

An ultra-high molar activity study with ^11^C-ITMM in rodents saw a significant increase in SUV and *V*_T_ compared with standard molar activity, highlighting molar activity as a potential risk for introducing variability [238]. A study investigating the relationship of ^11^C-ITMM *V*_T_ with age and gender reported increases in *V*_T_ in older controls [92]. However, due to significantly higher molar activity of tracer in the older population (average increase of 50% over young controls, *P*<0.01) and significantly lower injected activity, the *V*_T_ alterations quoted in the paper could be influenced by the lower injected dose of ITMM in older HC, in line with the pre-clinical work.

### Conclusions and outstanding issues for mGluR1 tracers

^11^C-ITMM and ^18^F-FIMX have shown excellent performance in pre-clinical characterisation studies for imaging of mGluR1. The higher uptake, faster kinetics, and longer half-life of ^18^F-FIMX suggest it will provide higher quality images and better quantification of mGluR1 in patients over ^11^C-ITMM, although further studies are required to verify this. Significant alterations of ^11^C-ITMM in ultra-high molar activity rodent studies highlight molar activity as a potential confound in clinical studies.

So far, ^11^C-ITMM has shown promise for cerebellar ataxia, where it has been shown to have a larger dynamic range than MRI methods and potentially more sensitive than ^18^F-FDG [90, 93]. However, no other disease states have been investigated with either tracer. Given the current interest in the glutamatergic system, studies are expected to emerge on a variety of disease states over the next few years.

## *κ* opioid receptor

In the brain, there are four subtypes of opioid receptors (OR), *μ*, *δ*, *κ*, and opioid-like receptor (OLR-1). Non-selective opioid PET tracers have been known and used in man for many years. However, for use in disease models and receptor occupancy studies, subtype selective tracers are required. For μ-OR, selective tracers have been well-characterised, with ^11^C-carfentanil commonly used clinically [239]. Additionally, *δ*-OR and OLR-1 selective tracers have been translated into humans, prior to the scope of this review [240, 241].

*κ*-ORs are the most abundant in the human brain [242] and have been linked to multiple disease states, including depression, stress, addiction, and pain response, as well as AD [243–247]. ^11^C-GR103545 and ^11^C-LY2795050 have recently been translated into man with the aim of selectively imaging *κ*-OR.

### Selectivity data for *κ*-OR tracers

Both ^11^C-GR103545 and ^11^C-LY2795050 showed promising in vitro selectivity over *μ*- and *δ*-OR [99, 248]. As an agonist tracer for *κ*-OR, ^11^C-GR103545 is expected to have low dose tolerance before negative pharmacological effects are observed. As such, self-blocking protocols are very limited, with changes in *V*_T_ values obtained in NHP too low for an accurate Lassen plot to be constructed [97]. Brain distribution of ^11^C-GR103545 was reported to align with expected *κ*-OR density in NHP [96]. Blocking with naltrexone showed substantial decreases in *V*_T_ in human studies; however, naltrexone is known to bind to *κ*-, *μ*-, and *δ*-OR [99, 249].

^11^C-LY2795050 is an antagonist tracer for *κ*-OR. In vivo blocking studies showed good response to both homologous block and heterologous block, with the non-selective opioid ligand naloxone reducing BP_ND_ to approximately 0 in NHP, showing approximately all specific binding signal is opioid-related [99]. Further studies in NHP were conducted where non-radioactive LY2795050 was used to block the signal of ^11^C-LY2795050 and *μ*-OR tracer ^11^C-carfentanil to determine the selectivity of LY2795050 for *κ*-OR over *μ*-OR. The in vivo results showed 7.6 fold selectivity for *κ*-OR over *μ*-OR, much lower than the reported in vitro selectivity of 36 fold [102].

### Pharmacokinetic profile of *κ*-OR tracers

In humans, the kinetics of ^11^C-GR103545 are slow, leading to complications in the calculation of kinetics and outcome parameters, compounded by the use of ^11^C. This will most likely be a substantial factor in the moderate-high degree of variability observed, with TRV on average 41% for the highest uptake region [96].

For ^11^C-LY2795050, the in-human pharmacokinetic properties observed are good. It had relatively high initial brain uptake and fast kinetics suitable for accurate kinetic modelling, although no reference region is available in humans [98]. Occupancy studies largely removed regional uptake differences showing almost full occupancy of receptor [98], and the test-retest repeatability was good [101].

### Conclusions and outstanding issues for *κ*-OR tracers

^11^C-GR103545 suffers from a number of drawbacks including lack of in vivo selectivity studies, due to target related toxicity and poor pharmacokinetics in humans. The binding affinity of ^11^C-GR103545 determined in vitro was very high, with a *K*_i_ of 0.02 nM [248]. As discussed by the authors of this work, a lower affinity derivative may have faster kinetics and be more suitable for imaging in vivo, highlighting that too high affinity can hinder kinetic analysis.

The moderate selectivity of ^11^C-LY2795050 for *κ*-OR over *μ*-OR in vivo is a manageable limitation of this tracer. In vivo, the vast majority of signal is expected to arise from *κ*-OR due to the moderate selectivity and higher natural abundance of *κ*-OR, especially in regions of high *κ*-OR to *μ*-OR density. ^11^C-LY2795050 appears the more promising of the two tracers due to its larger dynamic range of occupancy reporting and lower variability. However, experiments must be carefully designed in order to reduce, or control for, potential perturbation of signal by *μ*-OR alterations. There is therefore a scope for more selective tracers to make an impact on this field.

## Serotonin 5-HT_2_R

The serotonergic system is one of the major signalling pathways in the brain and as such is relevant to a broad range of disease states [250]. PET tracers have been evaluated in humans from four of the seven serotonin receptor sub-families (5-HT_1A_, 5-HT_1B_, 5-HT_2A_, 5-HT_4_, and 5-HT_6_) as well as the serotonin transporter (SERT) [251].

Imaging of the 5-HT_2A_R subtype is well established through antagonist tracers such as ^18^F-seperone and ^18^F-altanserine, with differences in receptor density identified in patients with obsessive-compulsive disorder [252], Tourette’s syndrome [253], schizophrenia [254], and AD [255]. However, while able to report on receptor density, 5-HT_2A_ antagonist tracers are unable to distinguish between the high- and low-affinity states of 5-HT_2A_ and are relatively insensitive to endogenous neurotransmitter concentration [256, 257]. As such abnormalities related to the 5-HT_2A_ high/low-affinity ratio or serotonin levels are unable to be identified using these tracers. The recent development and translation of ^11^C-Cimbi-36 represent the only clinical 5-HT_2_R agonist tested in man and with it the possibility of imaging high-affinity 5-HT_2A_ receptor.

### Selectivity data for ^11^C-Cimbi-36

In vitro characterisation showed high binding affinity to all three 5-HT_2_R subtypes (A–C) [258]. In NHP, selective blocking of 5-HT_2C_R was achieved with SB 242084 and showed approximately 100% decrease in BP_ND_ in areas of high 5-HT_2C_R density such as the choroid plexus [104]. In both human and NHP blocking, studies with 5-HT_2_R antagonist ketanserin (non-selective between 5-HT_2_ subcategories A–C) showed significant reduction across the brain (Table 3) [103, 104]. Pre-clinical studies have shown a significant response to pharmacologically increased and decreased levels of serotonin in vivo in NHP and rodents respectively showing the potential sensitivity of ^11^C-Cimbi-36 to endogenous neurotransmitter concentration [257, 259].

### Pharmacokinetic profile of ^11^C-Cimbi-36

In humans, the uptake of ^11^C-Cimbi-36 was relatively high in cortical regions although the kinetics were moderately slow. Metabolism of the tracer was relatively fast; however, the reported metabolites were more polar than ^11^C-Cimbi-36 and are not likely to cross the BBB. The lack of signal alteration upon blocking in the cerebellum highlights this as a suitable reference region, although a consistent negative bias was found when using this, and the human test-retest showed very low variability across brain regions [104].

### Conclusions and outstanding issues for ^11^C-Cimbi-36

^11^C-Cimbi-36 is the first 5-HT_2_R agonist PET tracer translated into man with pharmacokinetics acceptable for in-human imaging. While it appears not to be selective for 5-HT_2_R subtypes, the distribution of these within the brain is substantially different. 5-HT_2B_R is expressed in low concentrations in the human brain and is therefore unlikely to significantly contribute to observed signal [260]. For 5-HT_2A_R and 5-HT_2C_R, regional distribution differences may allow selective determination of subtype signal due to some areas of high 5-HT_2A_R being almost devoid of 5-HT_2C_R and vice-versa (i.e. cortical regions and choroid plexus, respectively). Therefore, in these regions, ketanserin can be treated as a pseudo-selective block in HC. However, caution must be expressed when interpreting results from regions of subtype colocalisation or disease states.

Further studies comparing agonist and antagonist tracers will hopefully provide a more comprehensive report on the role the 5-HT_2_ receptor plays in neuropsychiatric disorders.

## P2X7

The role inflammation plays in the initiation, progression, and symptoms of neuropsychiatric conditions is currently a key research question in medicine. However, imaging inflammation is not straight forward. Pro-inflammatory cells such as M1-activated microglia are upregulated at sites of inflammation and provide a range of possible targets [261]. One of the most successful strategies to date is tracers for translocator protein (TSPO) which is upregulated on activated microglia [262]. The most well-known is ^11^C-PBR28. While multiple studies have shown promising results, there are various confounds with TSPO imaging (e.g. reviewed in Marques et al. [263]). An in-depth discussion is beyond the scope of this review; however, some of the major drawbacks include common TSPO polymorphisms giving rise to distinct high/low-affinity binding groups, low concentration of TSPO even in neurodegenerative disease states, and poor selectivity between pro-inflammatory M1 microglia and anti-inflammatory M2 microglia [262]. These confounds have given rise to the emergence of other targets for inflammation, both receptor and enzymatic.

One such target is ionotropic purinoceptor P2X7. In vitro and in vivo work has shown upregulation of P2X7 in activated microglia, with P2X7 agonists increasing and antagonists decreasing the release of pro-inflammatory markers, in models of inflammation [262, 264, 265]. These results have led to the proposition that P2X7 may be a selective marker for the pro-inflammatory M1 subpopulation of microglia, over the anti-inflammatory M2 subpopulation [262, 266]. However, currently, there is little evidence to support the lack of expression of P2X7 on the M2 subpopulation, with multiple studies contradicting this hypothesis [267, 268]. Nevertheless, P2X7 remains a target of interest both for microglial imaging and for therapeutic indications.

PET tracers for P2X7 are under current clinical evaluation, representing the first in vivo P2X7 evaluation in human. One tracer, ^18^F-JNJ-64413739, has initial human data published in peer-review. In-human work is currently being carried out on other tracers including ^11^C-JNJ-54173717 (^11^C-JNJ717) and ^11^C-GSK1482160, which have human data presented at conference [108, 110], and ^11^C-SMW139, which is currently being evaluated in PD patients (www.clinicaltrialsregister.eu, EudraCT Number: 2018-000405-23).

### Selectivity data for P2X7 tracers

Pre-clinical selectivity data on the current P2X7 tracers is limited. No small animal selectivity studies are published for ^18^F-JNJ-64413739; however, slides on pre-clinical data are available online (www.nas.edu). While the data available appears promising, in-depth review of this must wait until peer-review publications are available.

^11^C-JNJ-54173717 and ^11^C-SMW139 have both been characterised with a viral vector model which causes expression of human P2X7 in one brain hemisphere of rats, with control viral vector injected into the other. Both tracers showed increased uptake in the hemisphere with human P2X7 expressing viral vector and homogeneity upon blocking with P2X7 antagonists [107, 109]. For ^11^C-JNJ-54173717, the block compound was structurally similar whereas for ^11^C-SMW139, this was structurally dissimilar. Further studies in NHP with ^11^C-JNJ-54173717 showed a substantial decrease in observed SUV upon self-block and blocking with chemically distinct P2X7 antagonist (JNJ-42253432) [109].

^11^C-GSK1482160 has been characterised in a lipopolysaccharide model of inflammation where *V*_T_ was significantly increased upon treatment, with 97% of the increased signal displaced in lipopolysaccharide and homologous block–treated animals [111]. The in vivo selectivity profile for ^11^C-GSK1482160 is currently lacking heterologous P2X7 blocking data. In vitro response to P2X7 expressing cells and correlation to expression of P2X7 and activated microglia in an ex vivo multiple sclerosis rodent model both increase confidence in selectivity [111, 112], but are not in themselves proof of P2X7 binding in vivo.

### Pharmacokinetic profile of P2X7 tracers

^18^F-JNJ-64413739, ^11^C-JNJ-54173717, and ^11^C-SMW139 appear to have relatively fast kinetics and high brain uptake suitable for imaging in the species investigated [106, 107, 109]. ^11^C-GSK1482160, however, has slow kinetics in rodent and NHP making accurate quantification more difficult. In rodents, ^11^C-SMW139 suffered from radiometabolites within the brain as well as limited dynamic range. Twenty-one percent of the brain radioactivity was observed as metabolites at 15 min, increasing to 34% by 45 min [107]. The presence of radiometabolites in the brain may be detrimental to the accurate quantification of P2X7 in human studies. ^11^C-JNJ-54173717 studies found no evidence of radiometabolites in rodent brain; however, upon self-blocking in NHP, an increase in SUV towards the end of the scan was observed, possibly indicative of brain penetrant metabolites, although this increase was not present in other protocols [109].

Human pharmacokinetics of ^18^F-JNJ-64413739 showed moderate TRV and high ICC suitable for PET imaging studies, although high COV indicates intrapatient variability may be high [106]. No data is available for metabolites of ^18^F-JNJ-64413739.

### Conclusions and outstanding issues for P2X7 tracers

The rapid translation of P2X7 tracers into humans signifies both academic and commercial interest in the area. Publication of pre-clinical and clinical data on these recently developed tracers will allow comparison and critical analysis and allow advancement of the quickly progressing P2X7 imaging field. For ^18^F-JNJ-64413739 and ^11^C-GSK1482160, the lack of published, heterologous blocking studies is a limitation in confidence going forward. Additionally, brain penetrant metabolites may be a constraint to one or multiple of the discussed tracers.

## Enzymatic targets

Dysfunction of enzymes within the CNS may substantially perturb neurochemical balance in neuropsychiatric and neurological disorders. From a therapeutic perspective, modulating enzymatic targets may allow more physiological modulation of neurotransmitter systems than alternatives such as receptor antagonists, with potentially lower risk of side effects and drug tolerance issues [269], making PET tracers for enzymatic targets highly desirable in drug development programmes. Table 4 outlines the recent tracers translated into man for enzymatic targets within the CNS.

**Table 4:** Parameters of PET radiotracers for new CNS enzyme targets in humans

Family	Target	Tracer	First in human	In vivo homologous block (parameter, species)	In vivo heterologous block (parameter, species)	Human TRV	Interpatient variability outcome: value (regions)	Highest uptake (parameter, region)	Reference region	Advantages	Limitations
Cox	Cox-1	^11^C-PS13	2018 (CA) [113]	−57% (*V*_T_, NHP) [114]	−55% (*V*_T_, NHP) [114]	Ongoing	N/A	N/A	N/A	Relatively good response by heterogeneous and homogeneous block, selectivity for COX-1 over COX-2 shown in vivo [114]. Only COX-1 tracer translated into man.	Limited data available. Low plasma free fraction and relatively high non-displaceable binding in NHP brain [114].
Mitochondrial complexes	MC1	^18^F-BCPP-EF	2018 (CA) [115]	N/A	−35% (SUV, rat) [116] -40% (*V*_T_, NHP) [117]	N/A	N/A	28 mL/cm^3^ (*V*_T_, striatum) [115]	N/A	First in class. High brain uptake, suitable kinetics in NHP [117]. Large dynamic range in vitro [118]. Significant response in multiple preclinical disease models [119–121].	Full blockade not available in vivo due to toxicity [116, 117]*.* No peer review in-human data published to date.
Histone deacetylases	HDAC 1-3	^11^C-Martinostat	2016 [122]	−80% (*V*_T_, NHP) [123, 124]	−35% (% uptake normalised to uptake at 6 min, rat) [123]	<10% [122]	COV, 11–19% [122]	18 mL/cm^3^ (*V*_T_, cerebellum) [122]	N/A	First in class for CNS quantification of HDACs. Robust pre-clinical characterisation [123, 124]. High brain uptake, significant response in schizophrenic patients. [125]	No reference region available. Slow kinetics, higher interpatient variability using V_T_ measurements.[122]
Phosphodiesterases	PDE2	^18^F-PF-05270430	2016 [126]	32% decrease in striatal *V*_T_ (NHP) [127]	N/A	≤10% [126]	ICC, 0.66–0.90 (all reported regions except putamen), 0.23 (putamen) [126]	1.3 mL/cm^3^ (*V*_T_, putamen) [126]	Cerebellum (not validated) [126]	Only in-human PDE2 tracer. Low TRV.	Low *V*_T_ and BP in humans [126]. Large non-specific binding in NHP brain [127]. Presence of metabolites in rat brain, cerebellum activity increased upon blocking [127]. No structurally diverse heterologous block in vivo.
	PDE10A	^18^F-JNJ42259152	2013 [128]	−89% (SUVr-1, rat) [129]	≈−100% (SUVr-1, KO mouse) [129]	<10% on average[130]	ICC, >0.85 [130]	1.0 mL/cm^3^ (*V*_T_, putamen) [130]	Frontal cortex [130]	Very good preclinical selectivity [129]. Significant response shown in HD patients [131]. Low TRV.	Low brain uptake [130]. Possible brain penetrating metabolite [130].
		^18^F-MNI-654	2014 [132]	N/A	N/A	20% [132]	N/A	4.5 mL/cm^3^ (*V*_T_, striatum) [132]	Cerebellum [132]	High *V*_T_ and BP_ND_ in humans [132].	Deemed inferior to ^18^F-MNI659 due to higher TRV, lower uptake and slower kinetics [132].
		^18^F-MNI-659	2014 [132]	N/A	−47% (*V*_T_, human) [133]	<10% [132]	ICC, >0.80 [132]	2.8 mL/cm^3^ (*V*_T_, globus pallidus) [132]	Cerebellum [132]	Suitable pharmacokinetics, fast kinetics, high BP_ND_ [132]. Significant response observed in HD [134, 135]. Low TRV.	Lack of pre-clinical in vivo selectivity data published.
		^11^C-IMA-107	2014 [136]	≈−100% (SUVr-1, pig) [136]	−67% (*V*_T_, NHP) [136]	12% [137]	COV, <10% (89 of 91 regions) [137]	2.2 (BP_ND_, putamen) [138]	Cerebellum [137]	Very good preclinical selectivity [136]. Suitable pharmacokinetics [136]. Significant response in HD and PD [138, 139]. Used on widest range of human disease states.	Lower BP_ND_ than other PDE10A tracers [136].
		^11^C-Lu AE92686	2014 [140]	−94% (*V*_T_, NHP) [141]	82% (RO, NHP) [140]	<10% [140]	COV, <20% [140]	6.5 (BP_ND_, putamen) [142]	Cerebellum [140]	High brain penetration, high BP_ND_ [140]. Significant response in schizophrenia patients [142]. Low TRV.	Cerebellum V_T_ has large errors with plasma input methods in NHP [141]. No cerebellum V_T_ data published in humans. Potential for brain penetrating metabolite. Slow kinetics [140].
		^11^C-T-773	2016 [143]	−80% (*V*_T_, NHP) [144]	−47% (*V*_T_, NHP) [145]	<10% [143]	ICC, >0.85 [143]	5.5 mL/cm^3^ (*V*_T_, putamen) [144]	None available	High brain penetration [143]. Low TRV.	Off-target specific binding in NHP and human brain preventing use of SRTM [143, 144].
Fatty acid amide hydrolase	FAAH	^11^C-CURB (URB694)	2013 [146]	−86% (SUV, Rat) [147]	>−90% (*λk*_3_, human) [148]	<10% on average[148]	ICC, 0.55–0.89 [148]	0.17 mL/cm^3^ (*λk*_3_, thalamus) [146]	None available	Very good response to blocking studies In human and rodent [147, 148]. Low TRV.	Irreversible kinetics, large brain background signal even with low *λk*_*3*_ values [146]. Possible affinity dependence on common FAAH polymorphisms [149].
		^11^C-MK-3168	2018 [150]	≈−50% (SUV, NHP) [151]	>−90% (V_T_, humans) [150]	<12% [152]	N/A	29 mL/cm^3^ (*V*_T_, undefined) [150]	None available	Reversible [152]. Very good response shown to blocking studies in humans and NHP [150, 151].	Slow kinetics, fast metabolism [150]. Decrease in signal upon blocking in proposed reference region [152].

*N/A* indicates no published data is available, *KO* is target knockout model. Values quoted for in vivo blocking studies represent the region of highest alteration observed in the greatest response protocol. TRV and ICC values represent all regions quoted in the corresponding literature unless stated. For specific method of ICC calculation, please refer to corresponding literature. Highest uptake value represents the largest average of the quoted parameter in reported regions in HC. Reference region quantification has been validated against full plasma input methodologies unless otherwise stated

## Cyclooxygenase-1

The inhibition of cyclooxygenase-1 (COX-1) and COX-2 enzymes underlies the anti-inflammatory response caused by a number of non-steroidal anti-inflammatory drugs, such as aspirin and ibuprofen [270]. They are present in the human brain in activated microglia and are, therefore, targets for inflammation imaging. No PET tracer has shown promise for COX-2 in human CNS to date. However, a very recent candidate for imaging COX-1 has emerged in ^11^C-PS13, which was the most promising candidate of a series of COX-1 tracers evaluated pre-clinically.

### Selectivity data for ^11^C-PS13

Self-block and blocking with Me-KTP-ME (prodrug selective for COX-1 over COX-2) in NHP both showed similar decreases in *V*_T_ (≈−55%), compared with baseline scans [114]. A negative control blocking study with a COX-2 selective compound (MC1) showed no significant alterations in uptake. Additionally, in vitro screening found *K*_i_ constants of >10 μM for a wide range of human recombinant receptors, indicating potential selectivity against those tested [114]. To date, no in-human data has been published in peer-reviewed journals; however, initial scans are currently being carried out in a clinical trial (www.clinicaltrial.gov, trial identifier: NCT03324646). Some of the early scan data has been presented at the Society of Biological Psychiatry conference (2018) [113].

### Pharmacokinetic profile of ^11^C-PS13

In NHP, ^11^C-PS13 had the highest brain uptake and *V*_T_ values of the series tested, as well as suitable kinetic profile, although there appears to be no suitable reference region [114]. The proportion of non-specific binding in the NHP brain is relatively high and free fraction in plasma low, reducing the dynamic range of the tracer. Early data presented on human brain distribution appears similar to that observed in NHP, although more comprehensive analysis will have to wait until peer-reviewed publication [113].

### Conclusions and outstanding issues for ^11^C-PS13

The most apparent disadvantage of ^11^C-PS13 is the high proportion of non-specific binding in the NHP brain. This is most likely related to the relatively high lipophilicity of ^11^C-PS13 (LogD=4.3). Therefore, future tracers may reduce this with less lipophilic derivatives. However, the data presented so far, while limited, appears promising and may mark the first successful ligand for COX translated into humans.

## Mitochondrial complex 1

Mitochondria play a crucial role as the major producers of adenosine triphosphate, ATP, in eukaryote cells. The largest of the proteins in this process is mitochondrial complex 1. Dysfunction in mitochondrial complex 1 has been linked to multiple disease states including PD, Leigh syndrome, and encephalopathy [271, 272]. Although other mitochondrial complex 1 tracers exist, such as ^18^F-Flurpiridaz used in cardiac imaging [273], only ^18^F-BCPP-EF has been successfully used for imaging of mitochondrial complex 1 in the brain.

### Selectivity data for ^18^F-BCPP-EF

Early pre-clinical characterisation of ^18^F-BCPP-EF showed moderate response upon dosing with selective mitochondrial complex 1 inhibitor, rotenone, in both rodents and NHP. However, due to the cardiac toxicity of rotenone, only very low doses were administered in vivo [116]. In vitro studies on rat brain slices allowed higher doses of rotenone and signal intensity of ^18^F-BCPP-EF was decreased by 96% upon maximum block, implying a larger dynamic range and greater selectivity than shown in vivo [118]. The moderate reduction of *V*_T_ in NHP upon blocking with rotenone may allow the estimation of *V*_ND_ and occupancy using a Lassen plot analysis, therefore giving greater information on dynamic range in vivo.

### Pharmacokinetic profile of ^18^F-BCPP-EF

High uptake and fast kinetics were observed in rat and NHP brain. Due to the ubiquitous nature of mitochondrial complex 1, no reference region is available for this target. Implementation of ^18^F-BCPP-EF for use in humans has recently been undertaken and 8 HC scanned. Initial data presented at conference showed high brain uptake with *V*_T_ values around 30 in the striatum [115].

### Conclusions and outstanding issues for ^18^F-BCPP-EF

^18^F-BCPP-EF is the first tracer to measure the availability of any mitochondrial complex within the CNS and may also have potential as a reporter for mitochondrial density. Since its discovery, multiple preclinical studies have used ^18^F-BCPP-EF and shown significant alterations in mitochondrial complex 1 availability in models of ischemic injury and PD [119, 121], as well as showing negative correlations with age and amyloid load in NHP [120]. However, no in-human patient data are currently available. The toxicity of mitochondrial complex 1 inhibitors is a drawback of the field, as full in vivo blockade is unfeasible.

## Histone deacetylase

The histone deacetylase (HDAC) family of enzymes are transcription regulators and frequently implicated in epigenetic mechanisms, biomedical processes which alter gene expression as a result of environmental interactions with an individual’s genome [124]. They have been implicated in multiple disease states both in the periphery and CNS, including AD, HD, cancers, and immune disorders, amongst others [274]. For imaging of HDACs in humans, multiple PET tracers have been developed; however, most are unsuitable for CNS imaging due to poor brain penetration or the presence of brain penetrating metabolites [275]. ^11^C-Martinostat is the only HDAC tracer evaluated in humans suitable for imaging HDAC within the CNS.

### Selectivity data for ^11^C-Martinostat

^11^C-Martinostat is reported to bind with high affinity to HDAC 1, 2, and 3 and to a lesser extent 6, from in vitro assays. In vivo self-block of ^11^C-Martinostat largely reduced *V*_T_ in NHP (Table 4), showing high level of specific binding in this species [123, 124]. In rodents, similar decreases in ‘% whole brain uptake normalised to uptake at 6 min’ was observed for both self-blocking at 2 mg/kg and pre-treatment of CN54, a structurally dissimilar HDAC inhibitor [123]. In vitro screening revealed that at 50 nM of Martinostat resulted in 24% inhibition of dopamine transporter, highlighting this as a potential off-target binding site. However, additional studies using dopamine transporter tracer ^11^C-β-CFT showed no alteration of signal upon dosing with Martinostat (1 mg/kg) showing that Martinostat does not compete to measurable quantities with ^11^C-β-CFT in vivo [123].

### Pharmacokinetic profile of ^11^C-Martinostat

^11^C-Martinostat has been shown in all investigated species to have high brain uptake, but slow kinetics [122–124]. Upon self-blocking protocol in NHP, ^11^C-Martinostat was decreased in all brain regions, highlighting the lack of reference region available [123]. In HC, intrapatient reproducibility via test-retest was high and the interpatient variability was moderately high for *V*_T_ measurements. Interpatient variability was improved using SUV_60-90_ as an outcome parameter; however, to be applied to patient populations would require validation against arterial input methods. In a comparison with ^11^C-Martinostat uptake in schizophrenic and schizoaffective cohorts compared with HC, SUVR was used as an outcome parameter. The activity in each region was referenced to the activity in the whole brain. This method showed significant alterations in schizophrenic patients in multiple regions; however, the use of SUVR in this way requires validation in patient populations with arterial input functions, as highlighted by the authors [125].

### Conclusions and outstanding issues for ^11^C-Martinostat

^11^C-Martinostat represents the first HDAC tracer to be useable for brain imaging in humans. The pre-clinical selectivity data for this compound appears robust, with good response to blocking protocols. The slow kinetics of the tracer is a disadvantage; however, for the purposes of determining brain penetration and occupancy of HDAC-targeted drugs, this tracer appears suitable. From the two published in-human studies, two different outcome parameters were proposed in SUV_60-90_ and SUVR (using the whole brain uptake as a reference region). Both of these methods would require further validation in patient populations. Standardisation of outcome parameter in future studies would allow direct comparison between results.

## Phosphodiesterase

Cyclic nucleotide phosphodiesterases (PDE) are intracellular enzymes which inactivate the secondary messenger molecules cyclic adenosine monophosphate (cAMP) and/or cyclic guanosine monophosphate (cGMP). Dysfunction of PDEs can impact multiple cell processes regulated by these messengers, including proliferation, metabolism, inflammation, and apoptosis [276]. As such, PDEs are of interest in many disease states, including schizophrenia, depression, and degenerative diseases [276, 277]. Of the 11 currently known PDE subtypes, pre-clinical PET tracers have been reported for PDE2, 4, 5, 7, and 10. Of these, tracers for PDE2, 4, and 10 have been assessed in man, with PDE4 tracers well-characterised prior to the scope of this review [278].

## PDE2A

PDE2A is one of three isoforms of PDE2 and is highly expressed in limbic structures and basal ganglia of the brain and is concentrated in glutamate synapses [127]. Pre-clinical data has shown links to synaptic plasticity and cognition with PDE2A inhibitors having being investigated predominantly as treatments for schizophrenia and migraine, but they are also of interest in degenerative diseases [279–281]. ^18^F-PF-05270430 is the only PDE2A-specific tracer, thus far, to be translated into man.

### Selectivity data for ^18^F-PF-05270430

In NHP, brain distribution concordant with PDE2A expression was found, and occupancy studies in NHP with PDE2A inhibitor PF-05180999 showed a decrease in *V*_T_ of 32% and a maximum BP_ND_ reduction of 72%, in high-binding regions (Table 4) [127]. However, in lower uptake regions (including the cerebellum as the reference region), *V*_T_ increased upon blocking, with no hypothesis from the authors discussed [127]. Due to high structural similarities, PF-05180999 would be expected to act as a homologous block. In vitro screening of PF-05270430 indicates selectivity over other PDE and CNS targets; however, no heterologous blocking studies have been reported with structurally diverse PDE2A ligands in vivo, reducing confidence in selectivity.

### Pharmacokinetic profile of ^18^F-PF-05270430

Pre-clinical characterisation ^18^F-PF-05270430 showed high uptake, fast kinetics, and low TRV in NHP [127]. In rodents, low levels of non-parent brain metabolites (5–7%) were observed indicating potential for BBB penetrant metabolites in clinic [127]. The occupancy and decrease in *V*_T_ reported indicate that the *V*_T_ signal is predominantly non-displaceable binding, restricting its dynamic range. Moreover, the metabolism of ^18^F-PF-05270430 varied substantially between NHP subjects. The percentage of ^18^F-PF-05270430 in plasma was very variable, ranging from approximately 10 to 45% across the three NHP subjects at 120 min post-injection in baseline scans. The percentage of ^18^F-PF-05270430 in plasma for intra-animal metabolism rate remained constant under test-retest conditions and upon blocking [127].

In the only published human data on ^18^F-PF-05270430, similar brain distribution to pre-clinical studies was observed, suggesting binding to PDE2 and low test-retest variability across brain regions reported [126]. For this study, the cerebellum was used as a reference region, although additional studies are required to validate this. Both *V*_T_ and BP_ND_ were low, decreasing dynamic range and reducing signal-to-noise ratios [126].

### Conclusions and outstanding issues for ^18^F-PF-05270430

From the data presented to date, ^18^F-PF-05270430 may be adequate for some applications; however, it appears non-optimal for imaging PDE2A in humans and is lacking critical selectivity data. Therefore, while ^18^F-PF-05270430 may represent the first PDE2A tracer to be translated into humans, there is the need to develop new, more specific tracers in this field.

## PDE10A

PDE10A is a dual substrate PDE, degrading both cAMP and cGMP. It is localised primarily in medium spiny neuron cells of the striatum and other basal ganglia structures, with negligible levels in other brain regions [276]. The last 5 years have seen the emergence of multiple tracers for PDE10A successfully translated into humans. The first, ^18^F-JNJ42259152, was published in humans in 2013 [128], with four more the subsequent year, ^18^F-MNI-654, ^18^F-MNI-659, ^11^C-IMA107, ^11^C-Lu AE92686, and lastly ^11^C-T-773 in 2016 [132, 136, 140, 143]. Since publication, these tracers have been used for the study of PDE10A in HD, PD, and schizophrenia [131, 138, 282].

### Selectivity data for PDE10A ligands

The selectivity data for the in-human tracers of PDE10A (Table 4) generally show a high degree of selectivity across tracers, with the exception of ^18^F-MNI-654, for which no data are available, and ^11^C-T-773 which shows substantial off-target, specific binding. Two blocking studies have been published with ^11^C-T-773 in NHPs, both using female rhesus monkeys. The first used structurally dissimilar, PDE10A selective, MP-10 which showed a moderate decrease in striatal region *V*_T_ (caudate −36%, putamen −47%), indicating binding to PDE10A in these regions, with little effect on other regions investigated including the cerebellum [145]. The second used a structurally related drug candidate (TAK-063) which showed large decreases in *V*_T_ across the whole brain (approximately 75% on average) [144]. The large reduction of *V*_T_ in areas of negligible PDE10A concentration with TAK-063 that showed no response when blocked with PDE10A selective compound MP-10 strongly indicates that ^11^C-T-773 has substantial off-target specific binding to the unknown site(s) across these regions. There is therefore no reference region for this tracer and interpretation of results is complicated by potential alteration in off-target site expression/availability.

^18^F-JNJ42259152 is a structural derivative of selective PDE10A ligand MP-10; studies using this inhibitor are expected to act as a self-block. In the mouse KO model used for ^18^F-JNJ42259152 characterisation, homogenous brain distribution was observed, giving confidence of a high level of selectivity in the mouse. All other tracers are structurally dissimilar to MP-10 and utilised this compound in selectivity studies across a number of species, showing promising results (Table 4). For ^11^C-IMA107, structurally related compound IMA102 was used assumed to act as a self-block.

### Pharmacokinetic profile of PDE10A tracers

Kinetic profiles of the PDE10A PET tracers were generally reported to be suitable for PET imaging in human. ^18^F-JNJ42259152 showed similar brain distribution as pre-clinical models; however, *V*_T_ and BP_ND_ values were globally low with max *V*_T_ approximately 1.5 in the putamen and <1 for other regions [130]. Contrasting to the other PDE10A tracers, the frontal cortex was used as a reference region due to consistently showing the lowest uptake across brain regions. For all remaining tracers, the cerebellum was validated as an appropriate reference region (Table 4), with the exception of ^11^C-T-773 for which no reference region is available.

^18^F-MNI-654 displayed slower kinetics, higher variability, and lower uptake than ^18^F-MNI-659 and was therefore deemed an inferior PDE10A tracer [132]. All other reported tracers showed low to moderate inter- and intrapatient variability where reported (Table 4).

From preclinical characterisation, two tracers have evidence of brain penetrant radiometabolites. ^18^F-JNJ42259152 showed high quantities of radiometabolite in brain tissue of rats (53% of cerebellum activity, 60 min), which could make quantification of this tracer difficult. However, the identified metabolite is not expected to bind to PDE10A and authors of human studies claim this does not impact the quantification of PDE10A with ^18^F-JNJ42259152 [130]. ^11^C-Lu AE92686 underwent fast metabolism in NHP, with slow increase in cerebellum *V*_T_ observed throughout the scan, indicating a brain-penetrating metabolite may be present in small quantities [141]. The cerebellum *V*_T_ increase over time has not yet been reported in human, preventing the assessment of the possibility of brain-penetrating metabolite accumulation.

Research in HC with^18^F-MNI-695 have investigated mapping PDE10A, showing a significant negative correlation of striatal BP_ND_ with age, as well as increased BP_ND_ in women over men [135, 283]. ^18^F-MNI-659 has also been successfully applied to receptor occupancy studies [133, 135].

### Disease state imaging with PDE10A tracers

The first investigation into PDE10A in human disease was a pilot study with ^18^F-JNJ42259152. Significant decrease in BP_ND_ (alongside MRI volume) was observed in the putamen and caudate nucleus of HD patients compared with controls [131]. No significant correlation with disease severity was observed, although due to the small sample size (*n*=5), this is unsurprising. BP_ND_ images published did, however, hint at a trend towards significance.

In HD, ^18^F-MNI-659 BP_ND_ of the basal ganglia structures was around 50% of HC and showed a strong inverse correlation to both genetic burden (burden of pathology) and clinical disease rating (motor subscale score) [134]. Additionally, while both studies had small cohorts, and further studies are required, these results imply PDE10A is a major feature in HD pathology. They also support the applicability of ^18^F-MNI-659 for PDE10A imaging in HD.

In a cohort of 12 early premanifest HD patients, predicted several years until onset, ^11^C-IMA107 BP_ND_ was significantly decreased in multiple areas, with the largest in the sensorimotor striatum (−33.0% caudate, −30.5% putamen) as well as substantial increase in motor thalamic nuclei (+34.5%) compared with HC [139]. Significant correlations between ratios of motor thalamic nuclei BP_ND_/striatal BP_ND_ compared with predicted disease onset [139]. The authors claim this to be the strongest correlation with risk of symptomatic HD conversion of any technique, and the earliest in vivo pathophysiological mechanism identified. No comment was published on correlation directly between striatal BP_ND_ and risk of symptomatic HD conversion. The motor thalamic nucleus has BP_ND_ values substantially lower than close proximity structures of the basal ganglia. As a region, this may, therefore, be susceptible to partial volume correction errors and show substantial differences dependent on the analysis method used. Nevertheless, this study correlates well with that published for ^18^F-MNI-659, discussed above, with PDE10A emerging as a potential therapeutic target and ^11^C -IMA107 as a suitable tool for tracking it in clinical trials. Longitudinal studies will, however, be required to assess the predictive power of PDE10A imaging in symptomatic conversion.

In 24 PD patients, a significant reduction in ^11^C -IMA107 BP_ND_ was found in multiple basal ganglia regions, with the largest decreases observed in caudate (−28.4%) and putamen (−25.5%) compared with that in HC [138]. Significant inverse correlations were found between ^11^C -IMA107 BP_ND_, compared with disease duration and severity of motor symptoms [138]. As the only published PET study of PDE10A in PD patients, this work provides substantial justification for further investigations into the role of PDE10A in PD.

In a study investigating the role of PDE10A in schizophrenia, ^11^C -IMA107 showed no significant difference between patients and HC in any brain region, with all average differences within the variability of the tracer from previous test-retest data [282]. In contrast, ^11^C-Lu AE92686 showed significantly lower BP_ND_ in the putamen and caudate nucleus compared with that in HC, but no correlation to symptoms was found [142].

### Conclusions and outstanding issues for PDE10A tracers

Multiple tracers appear suitable for imaging PDE10A in humans; therefore, the selection of lead tracer to use in studies is not straightforward.

No head-to-head studies have been carried out on the tracers in humans (except MNI-654 and MNI-659), so conclusions on the applicability of tracers are subjective. However, comparison of the HC data collected so far allows forerunners for clinical imaging purposes to be identified. Due to its slow kinetics and high variability, ^18^F-MNI-654 appears unsuitable for clinical imaging. ^11^C-T-773 has the highest quoted *V*_T_ in HC, fastest kinetics, and low test-retest variability. However, the presence of an unknown off-target binding site, and the unsuitability of the cerebellum as a reference region, may restrict use in preference of other available tracers. ^11^C-Lu AE92686 also shows high brain uptake and low variability in high PDE10A regions. The kinetics of ^11^C-Lu AE92686 are slower than ideal and the indication of a possible brain-penetrating metabolite, confounding cerebellum *V*_T_, is the drawback of this tracer. Of the remaining tracers, ^18^F-JNJ42259152, ^18^F-MNI-659, and ^11^C-IMA107 all have appropriate kinetics for clinical imaging, have suitable test-retest, and have successfully been applied for disease state imaging. Of the three, ^18^F-MNI-659 has the highest *V*_T_ and BP_ND_ values; however, it currently has no self-bock in vivo data available. ^18^F-JNJ42259152 and ^11^C-IMA107 both have convincing selectivity data from in vivo pre-clinical studies. The low brain uptake of ^18^F-JNJ42259152 results in low *V*_T_ values, potentially complicating quantification, although BP_ND_ values remain moderately high. The reverse is the case with ^11^C-IMA107 which shows moderate *V*_T_ values but the lowest BP_ND_ values of the in-human tracers discussed. None of the in-human tracers is without flaws; however, multiple tracers appear suitable for clinical studies, with the current forerunners being ^18^F-JNJ42259152, ^11^C-IMA107, and ^18^F-MNI-659.

## FAAH

Fatty acid amide hydrolase (FAAH) breaks down fatty acid ethanolamide signalling molecules across the brain. These molecules act on a variety of receptors, including cannabinoid and vanilloid (TRPV1) receptors. Inhibition of FAAH increases endogenous levels of fatty acid ethanolamide, increasing the activity of associated receptors, potentially without the observed side effects of direct agonists [269]. Clinical trials have been instigated to examine FAAH inhibitors as potential therapeutics for pain, addiction, and Tourette syndrome (www.clinicaltrials.gov). Two FAAH tracers have been translated clinically to aid in studies of this enzyme, one which binds irreversibly, ^11^C-CURB, and one reversibly, ^11^C-MK-3168.

### Selectivity data for FAAH tracers

Both FAAH tracers showed promising pre-clinical blocking profiles in pre-clinical models and humans. Ex vivo data from rats showed ^11^C-CURB had regional heterogeneity in accordance with the known distribution of FAAH with high uptake in the cerebral cortex, cerebellum, and hippocampus, compared with that in the hypothalamus [147]. A high degree of specific binding was found in the regions investigated upon blocking with cold reference compound (also known as URB694) and the highly structurally related compound URB597 [147]. In humans, studies with the structurally dissimilar and selective FAAH inhibitor PF-04457845 showed an excellent response, decreasing the outcome parameter, λ*k*_3_ (see further explanation below), by over 90% and removing heterogeneity of this parameter across the brain, giving further evidence to the selectivity of the tracer [148].

^11^C-MK-3168 is the other FAAH tracer to be evaluated in humans and binds reversibly. Pre-clinical data show good brain uptake and response to blocking with a compound of the same series, as well as high occupancy with the structurally dissimilar FAAH inhibitor JNJ-42165279 [150, 151]. Additionally, an occupancy study in humans, with JNJ-42165279, showed high FAAH occupancy, correlating with dose, and decreased the *V*_T_ of ^11^C-MK-3168 by over 90% in multiple brain regions [150].

### Pharmacokinetic profile of FAAH tracers

Both tracers showed high brain uptake and distributions in line with FAAH distribution and similar TRV. With the different binding pharmacokinetics, analysis methods and outcome parameters for the two tracers are substantially different. Modelling of ^11^C-CURB revealed an irreversible two-tissue compartment model (2-TCMi) gave the most accurate fit, giving the outcome parameter of scans as λ*k*_3_ [146]. Here, *k*_3_ is the rate constant into the irreversibly bound compartment and *λ* is *K*_1_/*k*_2_, equivalent to the equilibrium distribution volume of the ligand in the free and non-specifically bound compartment. ^11^C-MK-3168 has the more intuitive outcome parameter of *V*_T_ measurements, but suffers from slow kinetics at baseline and rapid metabolism in humans (<5% parent after 30 min), with no published data on the nature of the metabolites. However, from the occupancy data reported, this does not appear to cause issue. Originally, white matter was proposed as a reference tissue for ^11^C-MK-3168 but a decrease in white matter signal was associated with blocking (<15%) and poorer repeatability reported [152]. Additionally, no reference tissue method was used in the subsequent clinical trial employing ^11^C-MK-3168 [150]. These observations suggest that white matter is not a suitable reference tissue for ^11^C-MK-3168.

The binding of ^11^C-CURB has been found to be linked to variation in a common nucleotide polymorphism on the FAAH gene (C385A, associated with increased risk of addiction); the λ*k*_3_ of ^11^C-CURB was found to be significantly lower (−23%) in those with the A allele (A/C+A/A) than those with the C/C alleles [149]. While the authors suggest the change may represent a decrease in *B*_max_, the possibility of decreased ^11^C-CURB binding affinity has not been ruled out, especially as the polymorphism alters a nucleotide close to the catalytic site [149]. Either way, controlling for this polymorphism in further studies employing ^11^C-CURB will be necessary. A further study by the group investigated the effect of cannabis and found significantly lower (−14% ^11^C-CURB λ*k*_3_ values) in cannabis users vs HC, and a negative correlation with clinical impulsiveness scale assessments [284]. In both reported studies, one subject with the A/A polymorphism was included and had the lowest λ*k*_3_ value from each cohort, implying an A-allele dose-response may be present. For ^11^C-MK-3168, no work has been published on the effect of C385A polymorphisms on binding properties.

### Conclusions and outstanding issues for FAAH tracers

These two tracers represent the first successful strategies to image FAAH in humans, proving feasibility of PET studies for this target. While the kinetics of ^11^C-MK-3168 are relatively slow, reversible tracers are preferred as quantification is easier and outcome parameters are more intuitive than their irreversible counterparts, such as for ^11^C-CURB. Nevertheless, both tracers have shown repeatability of their outcome parameters in humans and appear suitable for occupancy studies. The effects of the C385A polymorphism are a complicating factor for ^11^C-CURB studies; however, characterisation of the polymorphism impact will now be expected for all FAAH tracers.

## Overall limitations and future directions

Substantial progress has been made in multiple areas; however, many important targets have no suitable tracers in human. Misfolded proteins, such as α-synuclein which generates high interest in PD research and transactive response DNA-binding protein (TDP-43), emerging as a key molecular target in amyotrophic lateral sclerosis (ALS) and frontotemporal dementia [285], have proven to be difficult targets for radioligand development. Glutamate is the major excitatory neurotransmitter within the CNS, yet despite some success in the development of metabotropic glutamate receptor PET ligands, there are no useful radioligands for the major inotropic glutamate receptors, including direct targeting of AMPA, or any NMDA or kainate subtypes. Likewise, for γ-aminobutyric acid (GABA), the most widely distributed inhibitory neurotransmitter in the CNS [286], the majority of subunits of GABA_A_R (the major type found in the CNS) are lacking selective PET tracers, with none available for GABA_B_R or GABA_C_R.

Tracers able to image different aspects of targets are also desirable. Investigating the downstream effects of drugs on other CNS systems, by imaging of targets other than those directly bound by the drug, is becoming increasingly important to unpick the complex cascade processes within the brain [287]. The availability of PET tracers responsive to the receptor occupancy of endogenous neurotransmitters allows these effects to be explored, as has been the case for dopamine [288, 289]. Recent developments have provided evidence for the feasibility of imaging endogenous opioids [290], acetylcholine [291], and 5-HT [292] and this is an area that has the potential of expanding into other neurotransmitter systems. Allosteric modulators are an area of high interest in drug development programmes, with candidates binding away from the active site of the target. These in themselves provide distinct binding sites for PET tracers, enabling characterisation of target and allosteric ligand binding.

The development of PET tracers for new targets is challenging, with intensive screening processes required in development phases. Drug development campaigns in industry generate large compound libraries with associated screening data not available in the literature. These data provide important information on selectivity for PET tracer development programmes. Thus, one strategy to aid efficient PET tracer development could be precompetitive partnering with industry and academia to share data to identify and characterise the best tracers. This, in turn, could provide valuable new PET tools for industry to use in development and clinical trial programmes

## Overall conclusions

Our review identified 40 new tracers across 16 different CNS targets available for imaging in humans. The high number of CNS PET tracers for tau, P2X7, and PDE10A translated into humans within this timeframe shows the impetus for developing imaging tools to explore new targets in vivo. The selectivity and pharmacokinetic data we have summarised allow tracers to be compared and informed choices on which tracer to be selected for future studies made, as well as identifying information that needs to be determined to fully characterise some tracers. Many of the discussed tracers have the potential to make substantial contribution to the characterisation of diseases and drug development programmes, with some already having multiple in-human results published.

There are also, however, a number of recurrent issues that emerge throughout the review.

### Lack of pre-clinical in vivo blocking studies

Of the 40 tracers discussed, less than half have robust pre-clinical blocking studies published (defined as analogous *and* heterologous block in vivo corresponding to high RO). In vitro selectivity profiles, while useful for screening, are insufficient to prove in vivo selectivity but are repeatedly quoted as proof of tracer selectivity. Multiple tracers discussed have been used in clinical studies where the validity of findings may therefore be compromised due to the lack of characterisation, as seen in the tau imaging field.

### High and consistent molar activity

It is well known that ‘low’ molar activity will cause occupation of the target site due to self-block, effecting outcome parameters. However, the quantity of injected mass that would cause a non-negligible self-block is dependent on multiple factors including the target density and affinity of tracer for the target and is often not well characterised in humans. Therefore, the validity of results from studies using poor molar activity, or significantly different injected masses between groups, may be questioned, reducing confidence in results.

### Characterisation of off-target sites

Multiple tracers discussed have evidence of off-target binding sites. For some, such as ^11^C-LY2795050 and ^11^C-Cimbi-36, off-target sites are known and characterised, allowing assessment of when this limitation will and will not impede study outcomes. For others, however, the off-target site(s) are unknown, preventing informed decisions on potential outcome parameter impact, raising concerns on biological outcomes, and ultimately reducing tracer usefulness.

Addressing these issues will increase the chances of developing useful PET tracers for the CNS and help prevent late-stage issues arising. Notwithstanding these issues, the large number of tracers for new CNS targets identified in this review indicates there is considerable potential to advance understanding of brain function across disorders and informs the development of new therapies.
